# Microbial Degradation of Cellular Kinases Impairs Innate Immune Signaling and Paracrine TNFα Responses

**DOI:** 10.1038/srep34656

**Published:** 2016-10-04

**Authors:** Kenneth Barth, Caroline Attardo Genco

**Affiliations:** 1 Department of Integrative Physiology and Pathobiology, Tufts University School of Medicine, 136 Harrison Avenue, Boston, MA 02111, USA.

## Abstract

The NFκB and MAPK signaling pathways are critical components of innate immunity that orchestrate appropriate immune responses to control and eradicate pathogens. Their activation results in the induction of proinflammatory mediators, such as TNFα a potent bioactive molecule commonly secreted by recruited inflammatory cells, allowing for paracrine signaling at the site of an infection. In this study we identified a novel mechanism by which the opportunistic pathogen *Porphyromonas gingivalis* dampens innate immune responses by disruption of kinase signaling and degradation of inflammatory mediators. The intracellular immune kinases RIPK1, TAK1, and AKT were selectively degraded by the *P. gingivalis* lysine-specific gingipain (Kgp) in human endothelial cells, which correlated with dysregulated innate immune signaling. Kgp was also observed to attenuate endothelial responsiveness to TNFα, resulting in a reduction in signal flux through AKT, ERK and NFκB pathways, as well as a decrease in downstream proinflammatory mRNA induction of cytokines, chemokines and adhesion molecules. A deficiency in Kgp activity negated decreases to host cell kinase protein levels and responsiveness to TNFα. Given the essential role of kinase signaling in immune responses, these findings highlight a unique mechanism of pathogen-induced immune dysregulation through inhibition of cell activation, paracrine signaling, and dampened cellular proinflammatory responses.

The nuclear factor-kappaB (NFκB) and mitogen activated protein kinase (MAPK) signaling pathways are critical components of innate immunity that tailor appropriate immune responses to control and eradicate pathogens^1^,^2^. Detection of conserved motifs expressed by bacteria, fungi, and viruses occurs through binding to pattern recognition receptors (PRR) and leads to downstream autocrine and paracrine signaling through cytokine receptors, such as tumor necrosis factor-receptor 1 (TNF-R1), and function to amplify immune signals^3^. Upon recognition, immune signal activation initiates gene expression programs that promote inflammation and is reliant on kinase activity for proper initiation and propagation of proinflammatory signals^4^. Receptor-interacting protein kinase-1 (RIPK1) is a promiscuous regulator that integrates extracellular and intracellular stress signals, which controls cell activation, inflammatory signaling, and cell death-inducing processes^5^,^6^,^7^,^8^. The C-terminal domain of RIPK1 mediates TNF-R1-induced cell activation, apoptosis and necroptosis, by initiating interactions with TNF-R1-associated death domain protein (TRADD), Fas-associated death domain (FADD), or RIPK3, respectively. Upon TNFα stimulation, ligand binding induces trimerization of TNF-R1 and formation of a membrane-bound complex, which recruits TRADD, and then serves as an assembly complex for RIPK1, TRAF2, and ubiquitin ligases cIAP1/2^5^. RIPK1 becomes rapidly polyubiquitinated by K63 ubiquitin chains, which mediates the recruitment of transforming growth factor beta-activated kinase 1 (TAK1) and IKK complexes through ubiquitin binding, resulting in propagation of signal via kinase activity, phosphorylation of IκBα and subsequent activation of NFκB^5^. RIPK1 and TAK1 additionally can interact and activate MEKK3^9^,^10^,^11^, an upstream regulator of MAPK and NFκB. The key serine/threonine kinase regulator AKT is similarly induced in response to TNFα^12^ and displays common but also divergent pathways that may also require RIPK1/TAK1 signaling. Cross-talk between AKT with NFκB or MAPK pathways exists, as AKT directly phosphorylates IKKα^12^ and interacts with MEKK3^13^. MEKK3 holds a central role in both AKT and RIPK1-dependent signals, functioning as a divergence point in fine-tuning activation to either NFκB or MAPK.

Although the oral pathogen *Porphyromonas gingivalis* has evolved the ability to proliferate within inflammatory environments^14^, it also actively suppresses host inflammatory processes. A number of studies have demonstrated the ability of bacterial cysteine proteases (gingipains) as being indispensable for pathological outcome within *in vivo* models^15^, in addition to altering the innate immune response. Selective targeting of cytokines, antibacterial peptides, complement, cell adhesion molecules, coreceptors or MAPK pathways leads to dysregulation of cytokine networks, cell activation/recruitment, opsonization/phagocytosis and neutrophil function, effectively subverting protective proinflammatory host responses^16^,^17^,^18^,^19^,^20^,^21^. These studies highlight a significant role for gingipains on disruption of innate immunity and suggest dysregulation of host cell signaling may be a critical factor in many of these evasion strategies.

We previously demonstrated that *P. gingivalis* degrades both RIPK1 and RIPK2 in endothelial cells and macrophages in a dose and fimbriae dependent manner^22^. Apoptotic stimuli or PRR agonism failed to induce cleavage and degradation was mediated primarily by the *P. gingivali*s lysine specific cysteine protease gingipain Kgp^22^. In the current study, we examined the functional downstream consequences of gingipain activity on innate immune signaling pathways and pathway intermediates in primary endothelial cells and defined how this dysregulation of intracellular kinases impacts TNFα-dependent responses. Our results describe for the first time the selective degradation of RIPK1, TAK1, and AKT by a bacterial pathogen. Utilizing defined bacterial mutants together with lysine-specific cysteine protease inhibitors, we demonstrate that Kgp was solely responsible for the reduction in kinase protein abundance and resulting dysregulation of innate immune signaling, in addition to attenuation of TNFα dependent responses. Kgp was also shown to degrade mature cytokines and chemokines. Given the essential role of kinase signaling in immune responses, these findings highlight a novel pathogen mechanism of immune dysregulation via impairment of innate immune signaling and paracrine TNFα responses.

## Results

### Dampened Endothelial Response is Mediated by the Gingipain Kgp

**Figure 1:**
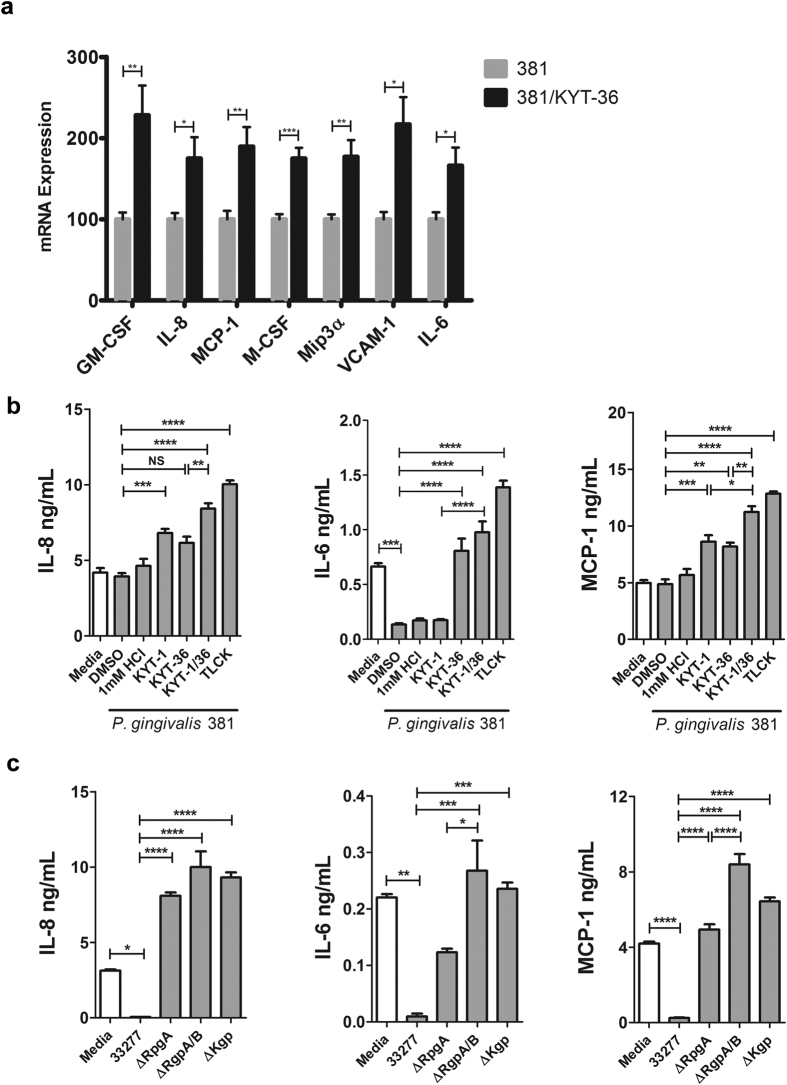
*P. gingivalis* gingipain activity suppresses bacterial-induced inflammatory mediator expression. (**a**) HUVEC were left untreated (media) or cocultured with *P. gingivalis* MOI 100 (381 or 381/KYT-36) for 8 hr. mRNA expression of proinflammatory mediators was assessed by qPCR. Values represent mean + SEM (n = 6) where 381 stimulated condition was set = 100%. (**b**) HUVEC were left untreated (media) or cocultured at MOI 100 for 24 hr with strain 381 pretreated with 10 μM KYT-1, 10 μM KYT-36, 10 μM KYT-1 and 10 μM KYT-36, 1 mM TLCK, or vehicle controls for 45 min. (**c**) HUVEC were cocultured with strain 33277, ΔRgpA, ΔRgpA/B, ΔKgp at MOI 100 for 24 hr. (**b,c**) Cell culture supernatants were examined for IL-8, IL-6, or MCP-1 via ELISA. ****p < 0.0001, ***p < 0.001, **p < 0.01, *p < 0.05. qPCR analysis included student’s t-test comparing 381 versus 381/KYT-36. ELISA analysis included one-way ANOVA with Bonferonni post test.

To investigate the effects of Kgp on endothelial inflammatory mediator mRNA expression during stimulation with *P. gingivalis*, we cocultured human umbilical vein endothelial cells (HUVEC) with *P. gingivalis* wild-type strain 381. *P. gingivalis* stimulation induced a number of chemoattractants, proinflammatory cytokines, and vascular adhesion molecules (Table S1). Pretreatment of bacteria with a Kgp inhibitor (KYT-36) resulted in an even greater mRNA induction of GM-CSF, IL-8, MCP-1, M-CSF, Mip3α, VCAM-1 and IL-6 (Fig. 1a), suggesting that Kgp actively inhibits inflammatory gene expression.

Considering *P. gingivalis* expresses several gingipains selective for specific arginine and lysine residues^15^, we examined the effect of selective gingipains on immune activation at the protein level. HUVEC were cocultured with wild-type strains 381 or 33277, or strain 381 pretreated with vehicle control or gingipain-specific inhibitors, or isogenic gingipain mutant strains (33277 genetic background) for 24 hr. Inhibition of gingipain activity in bacterial cultures was confirmed by biochemical analysis as previously reported (data not shown)^22^. Stimulation with *P. gingivalis* strains 381 or 33277 resulted in detection of IL-8, IL-6, and MCP-1 at protein levels either similar to media control or at reduced levels (Fig. 1b,c), although mRNA expression for each respective gene was significantly induced following bacterial stimulation (Table S1). Pretreatment of bacterial strains with the Rgp inhibitor (KYT-1), significantly increased IL-8 and MCP-1 production, while inhibition of Kgp with KYT-36 increased production of all cytokines tested (Fig. 1b), suggesting *P. gingivalis* mediated proteolysis of IL-8, IL-6 and MCP-1. Treatment with the general cysteine protease inhibitor, TLCK, or with both KYT-1 and KYT-36, which specifically block Rgp and Kgp activities, resulted in the greatest production of IL-8, IL-6 and MCP-1 (Fig. 1b), implying that *P. gingivalis* utilizes both Rgp and Kgp to degrade these cytokines. Similarly, infection with isogenic gingipain mutant strains (ΔRgpA, ΔRgpA/B, ΔKgp) resulted in significantly higher IL-8, IL-6 and MCP-1, compared to stimulation with gingipain-sufficient strain 33277 (Fig. 1c). We observed higher IL-6 and MCP-1 levels after stimulation with the *P. gingivalis* ΔRgpA/B strain compared to the *P. gingivalis* RgpA deletion strain, supporting a role for both RgpA and RgpB in degradation of IL-6 and MCP-1 (Fig. 1c).

### Kgp Degrades Innate Immune Signaling Proteins

**Figure 2:**
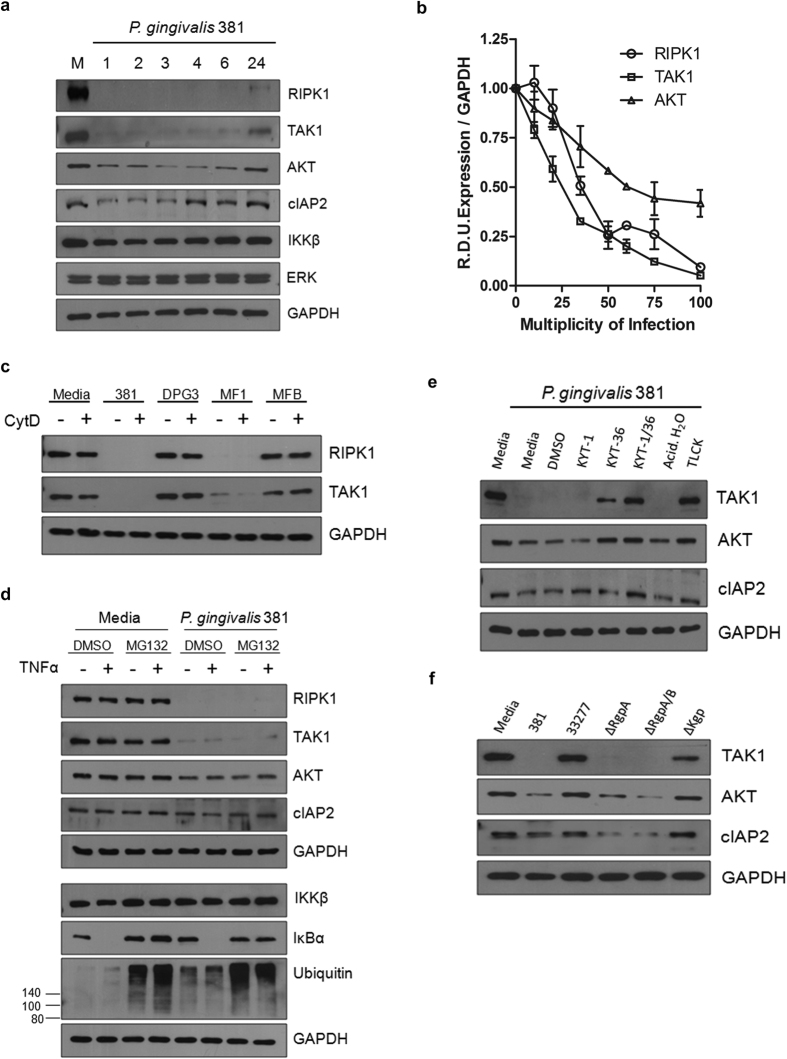
*P. gingivalis* degrades TAK1 and AKT and proteolysis is dependent on Kgp activity and major fimbriae. (**a**) HUVEC were left untreated (M: media) or cocultured with 381 MOI 100 for 1, 2, 3, 4, 6, or 24 hr. (**b**) HUVEC were cocultured with 381 MOI 10, 20, 35, 50, 65, 75, or 100 for 2 hr. Densitometry was performed on three independent membranes to calculate RIPK1 R.D.U. relative to GAPDH loading control. (**c**) *P. gingivalis* 381 or fimbriae mutant strains (major fimbriae mutant -DPG3, minor fimbriae mutant -MF1, or major and minor fimbriae mutant -MFB) were pretreated with DMSO or cytochalasin D (1 μg/mL) for 30 min and then immediately cocultured with HUVEC at an MOI 100 for 2 hr. (**d**) HUVEC were pretreated with vehicle control (DMSO) or MG132 for 2.5 hr (30 μM) and then left untreated or cocultured with *P. gingivalis* 381 for 2 hr, followed by restimulation with 10 ng/mL TNFα for 15 min. (**e**) *P. gingivalis* was pretreated with selective gingipain inhibitors (Rgp-specific: 10 μM KYT-1, Kgp-specific: 10 μM KYT-36, 10 μM KYT-1 and 10 μM KYT-36), the general cysteine inhibitor TLCK (1 mM), or vehicle controls for 45 min. HUVEC were then immediately cocultured with medium or pretreated preparations of *P. gingivalis* for 2 hr. (**f**) HUVEC were cocultured with wild-type strains 381, 33277, or gingipain mutant strains (ΔRgpA, ΔRgpA/B, ΔKgp) at an MOI 100 for 2 hr. (**a–f**) Whole cell lysates were analyzed for RIPK1, TAK1, cIAP2, IKKβ, P- IKKβ, IκBα, P-IκBα, AKT, ubiquitin, ERK, or GAPDH via Western blot analysis.

We previously reported that *P. gingivalis* mediated degradation of RIPK1 in human endothelial cells occurred early after stimulation with consistent generation of lower molecular weight fragments (sizes 14, 28, 32kDa) that were not products of new protein synthesis^22^. Considering immune signaling occurs through multiple pathways and mediators, we sought to examine if additional members of signaling nodes were affected by stimulation with *P. gingivalis*. TAK1 is a kinase integral to IL-1, TLR and TNFα signaling pathways. Similarly to that observed for RIPK1, TAK1 levels were significantly reduced early after stimulation with *P. gingivalis* (Fig. 2a). Other mediators of immune signal propagation, cellular inhibitor of apoptosis 2 (cIAP2) and AKT, demonstrated reduced protein abundance at earlier times points, although to a lesser extent (Fig. 2a). We observed a similar transient reduction in protein abundance of PI3K (p85), an important modulator of AKT activity during *P. gingivalis* culture (data not shown). Notably, cleavage of TAK1 and AKT correlated with the presence of lower molecular weight fragments (40, 44kDa and 36, 42kDa, respectively; data not shown), similar to observed effects on parental RIPK1. Expression of IKKβ was relatively unchanged following stimulation with *P. gingivalis*, while ERK and GAPDH served as sufficient loading controls at all time points. We observed dose-dependent degradation of RIPK1, TAK1 and AKT (Fig. 2b) as well as a dependence on major, but not minor fimbriae, which are surface expressed proteins known to be critical for initial host cell attachment and invasion (Fig. 2c)^23^. Pretreatment with cytochalasin D, which prevented actin polymerization and blocked bacterial invasion, had no effect on degradation (Fig. 2c), suggesting that degradation events were independent of bacterial-driven cell entry. Although wild-type strain 381 was most effective at reducing RIPK1, TAK1 and AKT protein abundance, other wild-type strains induced significant protein level changes within 2 hr of coculturing (RIPK1 degradation was 81%, 30% and 39%, respectively), with the exception of limited modification of AKT protein abundance by strains 33277 and A7436 (Supplementary Fig. 1).

We next investigated if host proteasome activity was responsible for the reduction in host protein abundance considering its role in degrading ubiquitinated proteins during NFκB activation. Pretreatment with the MG132 proteasome inhibitor prior to coculturing with *P. gingivalis* caused a significant accumulation of ubiquitin and a reduction in IκBα degradation during TNFα restimulation, confirming proteasome inhibition (Fig. 2d). MG132 treatment did not affect RIPK1, TAK1, or AKT degradation during coculturing with *P. gingivalis* (Fig. 2d), suggesting that reduction in protein abundance was not due to cell-intrinsic proteasome activity. mRNA expression of these signaling mediators was unaffected during coculturing with *P. gingivalis* 381 or with Kgp inhibition, suggesting that changes in protein expression were not due to decreases in transcript levels (data not shown).

To determine if gingipains were responsible for alterations in TAK1, AKT, and cIAP2 protein levels, stimulations were carried out using a gingipain inhibitor or gingipain deletion strains. Decreases in protein levels of TAK1, AKT, and cIAP2 were dependent specifically on Kgp, as inhibition of Rgp activity was ineffective at inhibiting degradation (Fig. 2e,f). Incubation of HUVEC with gingipain inhibitors alone had no effect on protein abundance (data not shown).

**Figure 3:**
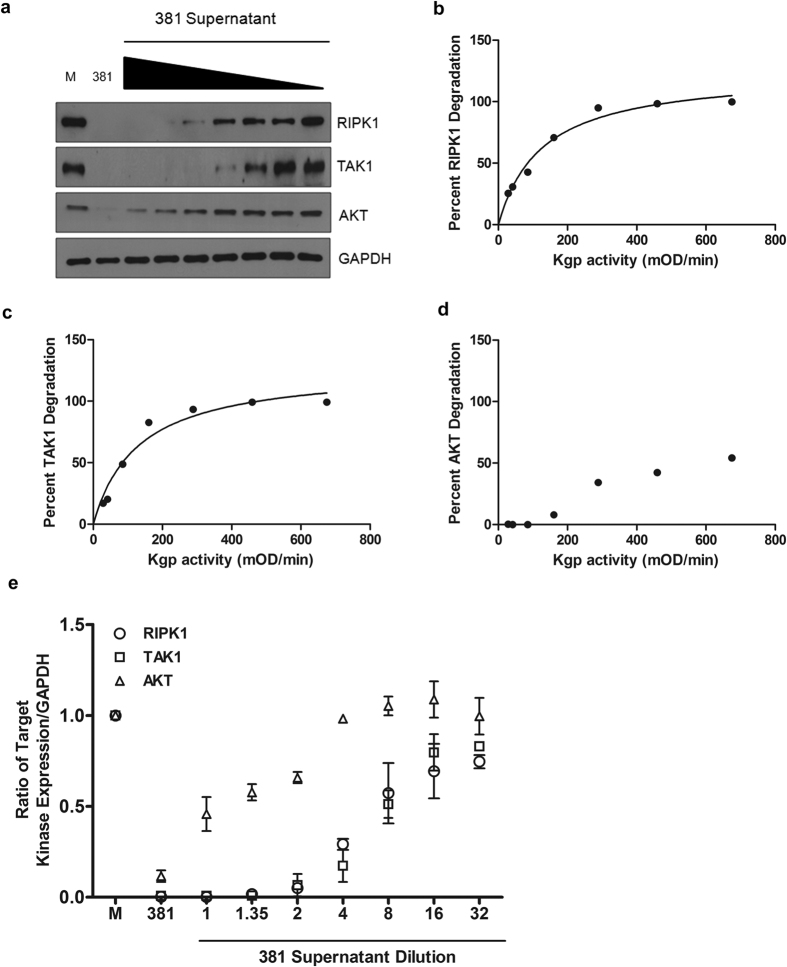
*P. gingivalis* proteolytic activity selectively targets RIPK1 and TAK1 with similar efficiencies. Bacterial supernatants of overnight grown broth cultures (1x equivalent to supernatant harvested from OD_660_ = 1 culture) were diluted 1.35, 2, 4, 8, 16, or 32x. (**a**) HUVEC were left untreated (M: media), cocultured with either *P. gingivalis* strain 381 MOI 100 (381), or incubated with bacterial supernatants harboring a range of decreasing Kgp-X activity (381 supernatant) for 2 hr. Whole cell lysates were collected and analyzed for RIPK1, TAK1, or AKT expression compared to GAPDH loading control. (**b–d**) Each bacterial supernatant was assayed for lysine-specific proteolytic activity by measuring the hydrolysis kinetics of N-Tosylglycl-L-prolyl-L-lysine-4-nitroanilide. Supernatant Kgp-X activities were calculated at 1.44, 2.07, 4.26, 8.04, 14.43, 22.97, and 33.75 mOD/min, respectively. Densitometry was performed on three independent Western blots in order to calculate the average percent degradation of (**b**) RIPK1, **(c)** TAK1, and **(d)** AKT, compared to Kgp-X activity. A Michaelis-menton model was used to calculate the enzyme activity required to degrade 50% of the host protein. (**e**) Protein abundance of RIPK1, TAK1, and AKT was calculated relative to GAPDH and plotted against untreated (M), MOI 100 (381), or a range of *P. gingivalis* dilutions and is shown as the ratio of target expression mean + SEM (n = 3).

To determine the efficiency of Kgp activity for each target, we cultured HUVEC with bacterial supernatants containing known proteolytic activity, and monitored protein degradation (Fig. 3a). Kgp efficiency was calculated by plotting percent degradation versus proteolytic activity. Kgp selectively degraded RIPK1 and TAK1 at similar efficiencies (50% of each protein was degraded with 126.3 or 130.1 mOD/min activity, respectively, Fig. 3b,c). In contrast, even at the highest Kgp activity, only 50% of AKT was degraded (Fig. 3d). We observed different Kgp target-specific efficiencies as demonstrated by the ratio of target kinase protein abundance relative to Kgp activity (Fig. 3e). Collectively, these results demonstrate that *P. gingivalis*-dependent changes to RIPK1, TAK1, and AKT protein levels occurs through the activity of Kgp, requires major fimbriae, and is dose-dependent however the mechanism by which Kgp gains access to intracellular proteins remains undefined. Previous studies indicate that the majority of adherent bacteria actively invade endothelial cells, suggesting a highly efficient endocytic uptake that occurs at lipid raft regions^23^,^24^. These lipid raft regions are unique in their clustering of host cell signaling proteins, thus suggesting a possible mechanism by which Kgp activity may be concentrated and localized at sites of entry, allowing access to specific sets of intracellular signaling components (kinases and adapter proteins).

### Gingipain Kgp impairs endothelial cell responses to TNFα

**Figure 4:**
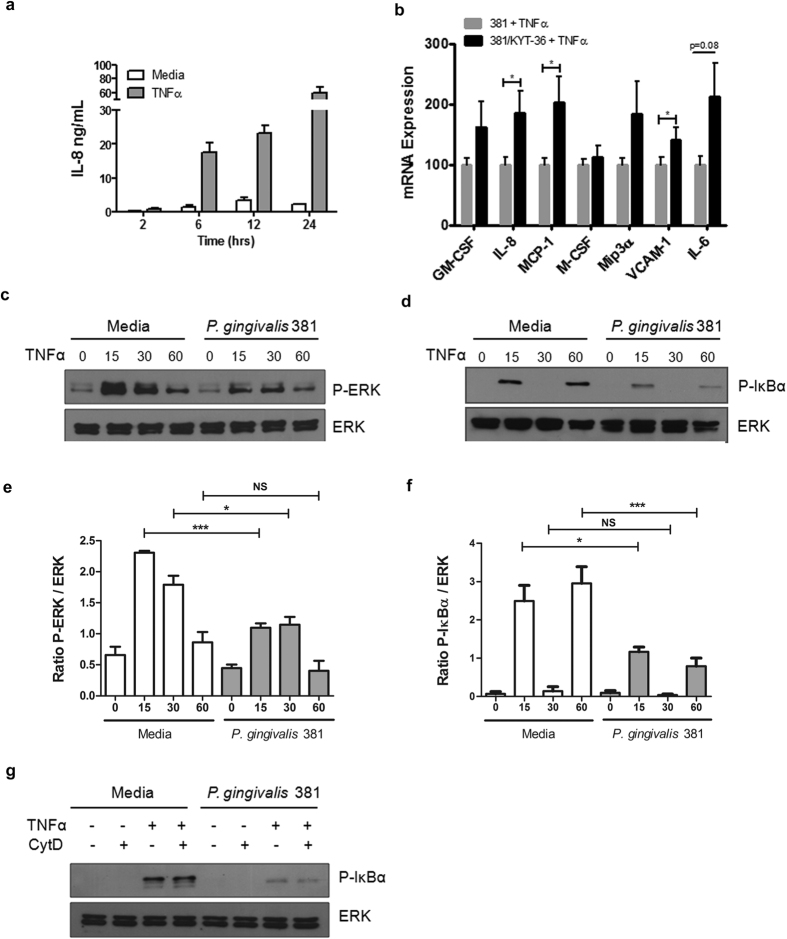
*P. gingivalis* stimulation of endothelial cells impairs TNFα-dependent signaling. (**a**) HUVEC were left untreated (media) or stimulated with 10 ng/mL TNFα for 2, 6, 12, or 24 hr and IL-8 expression was assessed by ELISA. (**b**) HUVEC were left untreated or cocultured with *P. gingivalis* MOI 100 (381 or 381/KYT-36) for 2 hr, followed by stimulation with 10 ng/mL TNFα for 6 hr. mRNA expression of proinflammatory mediators was assessed by qPCR. Values represent mean + SEM (n = 6) where 381 infected condition was set = 100%. qPCR analysis included student’s t-test comparing 381 versus 381/KYT-36, *p<0.05. (**c–f**) Cells were either left untreated (media) or cocultured with *P. gingivalis* (strain 381 MOI 100) for 2 hr, at which time cells were restimulated with TNFα (10 ng/mL) for 15, 30, or 60 min. Whole cell lysates were examined for expression of **(c)** P-ERK, or (**d**) P-IκBα. The ratio of (**e**) P-ERK to total ERK expression or the ratio of (**f**) P- IκBα to total ERK expression was quantified using densitometry from four independent Western blots. ***p < 0.001, *p < 0.05, NS = not significant, one-way ANOVA with Bonferroni post test. (**g**) HUVEC were pretreated with vehicle control (DMSO) or cytochalsin D (1 μg/mL) for 30 min prior to being left untreated or cocultured with *P. gingivalis* 381 MOI 100 for 2 hr. Cultures were then restimulated with 10 ng/mL TNFα for 60 min. Phosphorylation of IκBα was monitored via Western blot analysis.

Chronic inflammatory diseases caused by *P. gingivalis* are multifactorial, yet they share common features, including activated lymphocyte and macrophage infiltration at the site of inflammation. These recruited cells are capable of producing TNFα, an important initiator and modulator of chronic inflammation induced by this pathogen^25^,^26^. *Ex vivo* whole blood or splenocyte cultures stimulated with *P. gingivalis* induces significant TNFα, macrophages being the primary producer, however bacterial stimulated primary endothelial cells fail to induce TNFα secretion^26^,^27^ (Kenneth Barth, unpublished results). Within murine models of chronic inflammatory disease, TNFα is induced at the site of aortic lesions or within serum during oral lavage or injections of *P. gingivalis*, respectively^28^,^29^. Due to RIPK1’s central role in innate immune signaling pathways, especially those involving TNF-R, we next determined if *P. gingivalis* stimulated cells were impaired in their ability to respond to TNFα. As Kgp can degrade secreted cytokines and chemokines commonly used to measure immune cell activation (i.e. IL-8 and MCP-1; Fig. 1b,c) only signaling events downstream of cell activation (phospho-status) and inflammatory mRNA induction could be used to observe the effects of *P. gingivalis* protease Kgp on cell responsiveness to TNFα. Thus measuring secreted protein differences by ELISA would not discriminate between differences in cell activation that were due to decreased inflammatory gene induction or lack of secreted protein degradation due to the inhibition of Kgp activity. After 6, 12, or 24 hr of TNFα stimulation, we observed a significant induction of IL-8 production in HUVEC (Fig. 4a), demonstrating responsiveness to TNFα. We observed modest increases in proinflammatory mediator mRNA expression after TNFα stimulation of HUVEC cocultured with *P. gingivalis* strain 381 (Table S1). Pretreatment of bacteria with Kgp inhibitor KYT-36 led to increased mRNA expression of IL-8, MCP-1, and VCAM-1, compared to cells cocultured with bacteria in absence of inhibitor treatment (Fig. 4b). GM-CSF, Mip3α and IL-6 displayed similar expression profiles, but did not reach statistical significance. These results indicate that Kgp activity prevented TNFα-dependent inflammatory mediator induction.

### *P. gingivalis* Impairs TNFα-Dependent Activation of ERK and NFκB

To determine which signaling networks were responsible for dysregulated induction of proinflammatory mediators during TNFα stimulation of cells cocultured with *P. gingivalis,* we stimulated HUVEC previously cocultured with *P. gingivalis* with TNFα, and examined activation of MAPK (ERK, p38, JNK) or NFκB (IκBα) pathways. We observed significantly reduced levels of P-ERK and P-IκBα in cells cocultured with *P. gingivalis,* post-TNFα stimulation compared to stimulation in media control cells (Fig. 4c–f). Reduced RIPK1, TAK1, AKT, and cIAP2 protein levels were maintained throughout TNFα stimulation (data not shown). TNFα stimulation of HUVEC displayed a biphasic phosphorylation of IκBα (Fig. 4b), as observed previously^30^,^31^, while activation of p38 and JNK pathways was not impaired due to *P. gingivalis* (Supplementary Fig. 2). As with degradation of RIPK1, TAK1, AKT, and cIAP2, changes to IκBα phosphorylation did not require active bacterial invasion as pretreatment with cytochalasin D had no effect (Fig. 4e). Taken together, these results indicate that *P. gingivalis* infection impairs endothelial TNFα-dependent activation, resulting in specific impairment of NFκB and ERK pathways.

### Multiple Innate Immune Signaling Pathways Are Subverted by *P. gingivalis*

**Figure 5:**
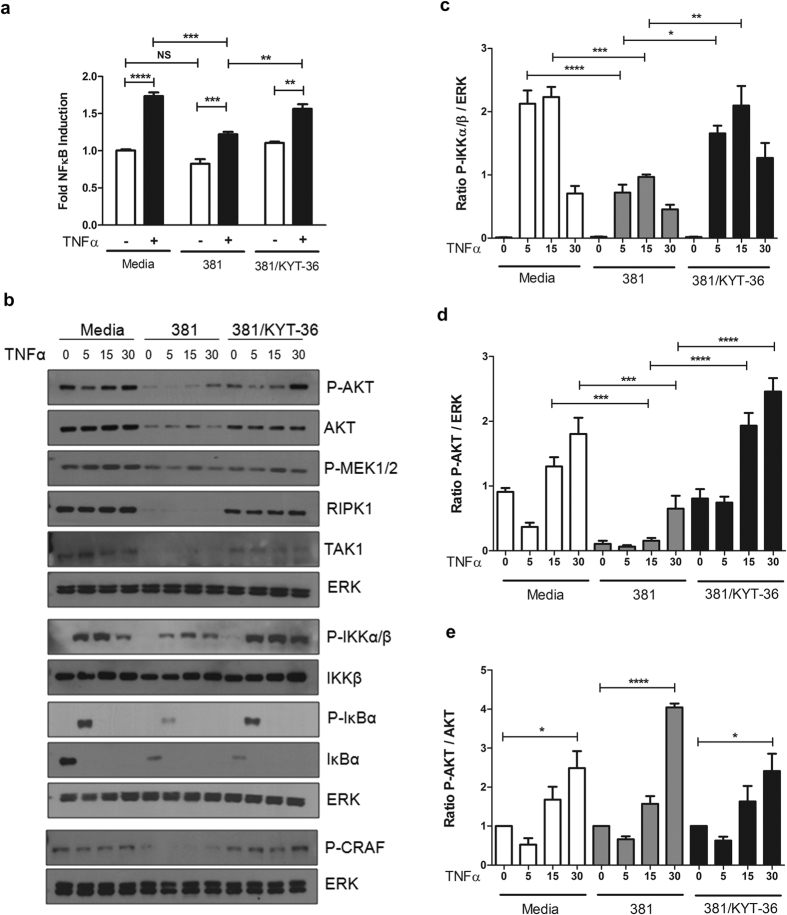
Impaired innate immune signaling occurs via alterations in AKT and IKKα/β activation and is dependent on Kgp activity. (**a**) HEK3293T cells were transfected with an NFκB-luciferase reporter and then left untreated, cocultured with *P. gingivalis* MOI 100 381, or 381 pre-incubated with 10 μM KYT-36 for 2 hr. Restimulation with 10 ng/mL TNFα was allowed to proceed for 6 hr, followed by NFκB activity analysis of whole cell lysates. Values represent fold NFκB induction relative to uninfected cells + SEM (n = 5), one-way ANOVA with Bonferroni post test. (**b**) HUVEC were left untreated (media), or cocultured with *P. gingivalis* MOI 100 (381 or 381 pretreated with 10 μM KYT-36) for 2 hr, followed by TNFα restimulation (10 ng/mL) for 5, 15, or 30 min. Whole cell lysates were collected and analyzed for activation status of a variety of signaling pathways downstream of TNF-R1 signaling, calculated via densitometry and depicted in (**c–e**). (**c**) The ratio of P-IKKα/β to total ERK expression (n = 3). (**d**) Ratio of P-AKT compared to total ERK (n = 3), where the ratio of P-AKT/ERK of time point zero for media was set = 1 R.D.U. (**e**) P-AKT induction was calculated for each condition (5, 15, and 30 min post TNFα restimulation). The induction values (P-AKT/AKT) are relative to the zero time point of no TNFα stimulation values for each respective condition (media, 381, 381/KYT-36). Values represent the mean + SEM (n = 4), one-way ANOVA with Bonferroni post test. For all panels ****p < 0.0001, ***p < 0.001, **p < 0.01, *p < 0.05, NS = not significant.

Considering innate immune signaling pathways have many nodes that act to converge/diverge signal propagation, we tracked potential impairment of both signal flux and terminal activation of ERK and NFκB pathways to further delineate the specificity of immune impairment. Using an NFκB reporter we observed TNFα-dependent activation of NFκB was impaired due to prior coculturing with *P. gingivalis* 381 (Fig. 5a). To track signal flux, phosphorylation of AKT, ERK (C-RAF, MEK1/2), or NFκB (IKKα/β, IκBα) pathway mediators following TNFα stimulation of cells cocultured with *P. gingivalis* was visualized by Western blot analysis. Cells cocultured with *P. gingivalis* displayed significantly reduced early phosphorylation of IKKα/β and IκBα at 5 and 15 min compared to media control (Fig. 5b,c). Similarly, AKT activation was dysregulated, with significantly reduced P-AKT/ERK expression also at 5 and 15 min post TNFα-stimulation in cells cocultured with *P. gingivali*s cells compared to media control (Fig. 5d). Notably, *P. gingivalis* infection alone induced significant reduction in total AKT protein abundance (Fig. 5b). Calculation of protein levels relative to total AKT (P-AKT/AKT) demonstrated that cells cocultured with *P. gingivalis* were equally capable of activating AKT compared to media control (Fig. 5e), although the magnitude was significantly decreased due to reductions in total AKT protein abundance. Analysis of kinases upstream of the ERK pathway revealed that coculturing with *P. gingivalis* alone greatly reduced C-RAF and MEK1/2 activation (Fig. 5b). Upon TNFα stimulation we confirmed abrogated signal flux of the ERK pathway in these cells as P-CRAF and P-MEK1/2 levels never reached activation levels compared to media control + TNFα (Fig. 5b). The observation that *P. gingivalis* both dephosphorylated and reduced total AKT, in addition to reducing CRAF and MEK1/2 activation, suggests that the AKT pathway is upstream of ERK-pathway members C-RAF and MEK1/2. Taken together, these results indicate that the presence of *P. gingivalis* results in the dysregulation of AKT, ERK, and NFκB pathways, indicated by impaired IKKα/β, IκBα, C-RAF, and MEK1/2 phosphorylation during TNFα responses.

To establish if impaired signal flux and NFκB activation were dependent on bacterial Kgp, comparisons were made between endothelial cells cocultured with Kgp-sufficient (381) and Kgp-impaired (381/KYT-36) bacteria. Inhibition of Kgp resulted in the recovery of endothelial responsiveness as demonstrated by increased NFκB activation and signal flux through NFκB and ERK pathways (Fig. 5a,b). TNFα-dependent phosphorylation of IKKα/β, IκBα, and AKT in endothelial cells cocultured with Kgp-impaired bacteria occurred at levels similar to media control (Fig. 5b). Similar recovery of AKT and IκBα phosphorylation was observed with restimulation of endothelial cells cocultured with strain ΔKgp (data not shown, Supplementary Fig. 3a,b). We observed no differences in degradation of IκBα between cells cocultured with strain 381 or Kgp-impaired bacteria (381/KYT-36, data not shown), suggesting that difference in P-IκBα were not due to depleted IκBα pools. These results indicate that *P. gingivalis*-dependent blunting of TNFα responses is driven by Kgp.

### Sustained Concentration and Bioactivity of TNFα During Restimulation Experiments

A number of reports investigating *P. gingivalis* gingipains have suggested that cytokines can represent substrates for proteolysis, therefore we examined if impaired TNFα-dependent signaling was a result of TNFα cleavage or proteolytic events that may affect its bioactivity^21^,^32^. Recovered TNFα concentrations were equivalent regardless of the presence of *P. gingivalis* or Kgp activity during coculture (Supplementary Fig. 4a,d). Notably, human endothelial cells failed to induce TNFα in response to bacterial stimulation, suggesting that all of the TNFα present within the *in vitro* system was from exogenous addition (Supplementary Fig. 4a,d). In addition, measurement of TNFα bioactivity in *P. gingivalis* stimulated cultures was similar to media control, regardless of Kgp activity (Supplementary Fig. 4b,c,e). Western blot analysis of concentrated supernatants from cell free incubation of recombinant TNFα with 3 × 10^7^ *P. gingivalis* (MOI100 equivalency used within stimulation experiments) displayed no apparent loss of detectable TNFα (data not shown). Thus *P. gingivalis* fails to alter exogenous TNFα concentrations or its bioactivity.

### TNFα-dependent signaling and inflammatory induction correlates with RIPK1, TAK1 and AKT protein abundance and/or activity

To determine if changes in RIPK1 expression correlated with the degree of cell responsiveness, we cocultured HUVEC with a range of *P. gingivalis* MOI and monitored IκBα phosphorylation. While cells cocultured with wild-type strain 381 exhibited a significant ablation of IκBα phosphorylation at MOIs of 70 and 100, we observed a trend of decreased P-IκBα expression with increasing concentrations of bacteria (Supplementary Fig. 5a,b). This trend was lost after treatment with a Kgp inhibitor or use of the ΔKgp strain (Supplementary Fig. 5a,b). These data suggest that increasing the amount of bacteria present initiates further reduction in cell activation to TNFα stimulation.

**Figure 6:**
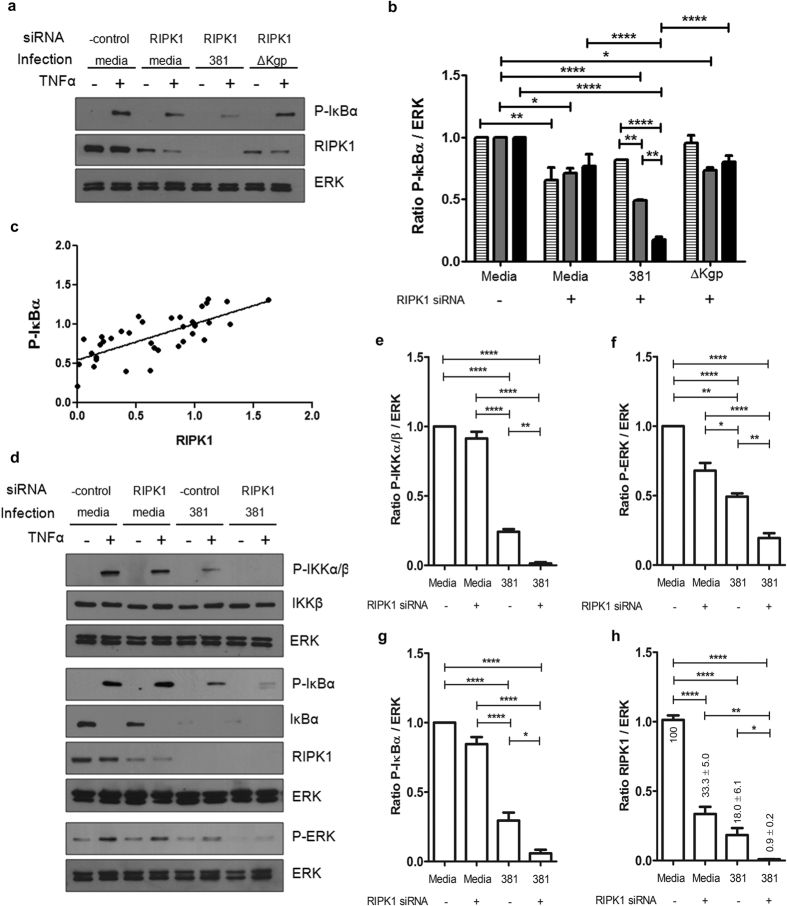
Endothelial RIPK1 protein abundance directly correlates with cellular responsiveness to TNFα. (**a**,**b**) HUVEC were transfected with either negative control or RIPK1 specific siRNA for 20 hr. Cultures were then left uninfected or cocultured with *P. ginigivalis* strain 381 or ΔKgp (MOI 35: hatched bars, 70: grey bars, 100: black bars) for 2 hr, followed by stimulation with TNFα (10 ng/mL) for 60 min in order to monitor cellular activation. (**a**) Whole cell lysates were probed for P-IκBα, RIPK1, and ERK. A representative blot for only MOI100 is depicted. (**b**) Densitometry was performed (n = 3) while comparing each of the three different multiplicities of infection (35, 70, 100) and the ratio of P-IκBα/ERK was calculated by setting negative control + TNFα = 1. ****p < 0.0001, **p < 0.01, *p < 0.05, two-way ANOVA with Bonferroni post test comparing each MOI across all conditions. (**c**) The relationship between P-IκBα and RIPK1 R.D.U. are graphed for each representative phosphoblot for TNFα stimulated samples at each MOI (35, 70, 100), with or without RIPK1 siRNA knockdown. A linear correlation with R_2_ = 0.5633 demonstrates that RIPK1 protein abundance dictates endothelial responsiveness to TNFα. (**d–h**) HUVEC were transfected with either negative control or RIPK1 specific siRNA for 20 hr, followed by coculturing with *P. gingivalis* 381 MOI 100 for 2 hr and then restimulation with TNFα (10 ng/mL) for 15 min. (**d**) Whole cell lysates were examined via Western blot analysis to determine TNFα-dependent activation status. Densitometry of independent membranes (n = 3–5) was used to determine (**e**) P-IKKα/β, (**f**) P-ERK, (**g**) P-IκBα, and (**h**) RIPK1 R.D.U. expression. RIPK1 protein abundance was calculated as % of media. ****p < 0.0001, **p < 0.01, *p < 0.05, one-way ANOVA with Bonferroni post test.

As further proof that loss of RIPK1 impairs inflammatory responses of endothelial cells to TNFα, we performed RIPK1 siRNA knockdown experiments. RIPK1 knockdown alone caused a modest reduction in cell responsiveness to TNFα (Fig. 6a,b). RIPK1 protein level was moderately reduced during RIPK1 siRNA treatment, but was severely attenuated during siRNA and *P. gingivalis* coculture in the presence of Kgp (Fig. 6a). RIPK1 knockdown alone caused a modest reduction in cell responsiveness to TNFα, however conditions of RIPK1 knockdown + *P. gingivalis* displayed significantly reduced P-IκBα, which was dependent on bacterial MOI (Fig. 6a,b), implying that a further reduction in RIPK1 protein abundance caused impaired cell activation. We observed a positive linear correlation between host RIPK1 protein level and P-IκBα (Fig. 6c), suggesting that the ability of endothelial cells to respond to TNFα was dependent on RIPK1 protein abundance. This was further confirmed as RIPK1 knockdown resulted in a significant reduction in mRNA expression of the downstream inflammatory mediators, GM-CSF, Mip3α, and VCAM-1, following TNFα stimulation (Supplementary Fig. 5c). To further understand the specific effect of RIPK1 knockdown on immune signal flux, knockdown or control cells were stimulated with TNFα after coculturing with *P. gingivalis.* RIPK1 knockdown alone resulted in no change or a slight decrease in IKKα/β, IκBα, and ERK phosphorylation after TNFa stimulation compared to media control (Fig. 6d–g). Similarly to previous results, TNFα stimulation of cells cocultured with wild-type strain 381 alone were significantly impaired in their ability to phosphorylate IKKα/β, IκBα, and ERK, which was further exacerbated by RIPK1 knockdown (Fig. 6d–g). RIPK1 protein abundance after siRNA treatment and *P. gingivalis* coculture was significantly lower than *P. gingivalis* coculture alone (Fig. 6d,h), suggesting that RIPK1 knockdown and *P. gingivalis* coculture have an additive effect on immune signaling impairment. Collectively, these data imply that *P. ginigivalis* dysregulation of TNFα immune signaling is mediated by attenuation of RIPK1 protein levels.

Given the involvement of TAK1 in NFκB-mediated inflammatory responses, we postulated that cell responsiveness to TNFα would be dependent on sufficient TAK1 protein abundance in addition to RIPK1. Coculture kinetics were used as a method to evaluate cell responsiveness as host cell kinase protein level changes occur transiently. RIPK1 and TAK1 abundance was severely attenuated early (2 hr), but recovered by 24 hr of stimulation with *P. gingivalis* (Supplementary Fig. 6a). Unlike cells cocultured with *P. gingivalis* wild-type strain 381 for 2 hr, cells cocultured for 24 hr displayed no changes in IκBα, IKKα/β, and ERK phosphorylation upon TNFα stimulation (Supplementary Fig. 6a,b). Furthermore, knockdown of RIPK1 and TAK1 resulted in reduced cellular signaling (P-IKKα/β, P-IκBα) compared to RIPK1 siRNA alone (Supplementary Fig. 7a–c) and rescue of cellular signaling within cells cocultured with *P. gingivalis* was achieved by overexpression of RIPK1 and TAK1 (Supplementary Fig. 7d,e). Basal activation levels of IKKα/β were increased under non-stimulated conditions compared to cells expressing endogenous levels of RIPK1 and TAK1, however the overall induction of IKKα/β phosphorylation was restored, suggesting that inflammatory signaling during coculture with *P*. *gingivalis* relies not only on RIPK1 abundance, but also TAK1.

Similar to the function of RIPK1, PI3K and AKT function to maintain NFκB activation during TNFα stimulation^12^. Previous studies suggest that AKT and RIPK1-dependent pathways include some degree of cross-talk^33^, as evidenced by interaction between AKT and RIPK1 to interact with or to activate MEKK3 and IKK^10^,^12^,^13^. Considering *P. gingivalis* infection reduced the abundance of AKT, we next examined if endothelial responses to TNFα could proceed through the PI3K/AKT pathway. Pretreatment of HUVEC with Ly294, a PI3K/AKT inhibitor significantly impaired cell activation, as measured by phosphorylation of IKKα/β and IκBα (Supplementary Fig. 8a) and this culminated in significantly impaired induction of inflammatory mediators GM-CSF, MCP-1 and VCAM-1 (Supplementary Fig. 8b). Collectively, these results indicate that RIPK1, TAK1 and AKT protein abundance and/or activity dictates endothelial cell responses to TNFα, and thus play a central role in bacterial-induced dysregulation of TNFα signaling.

## Discussion

**Figure 7:**
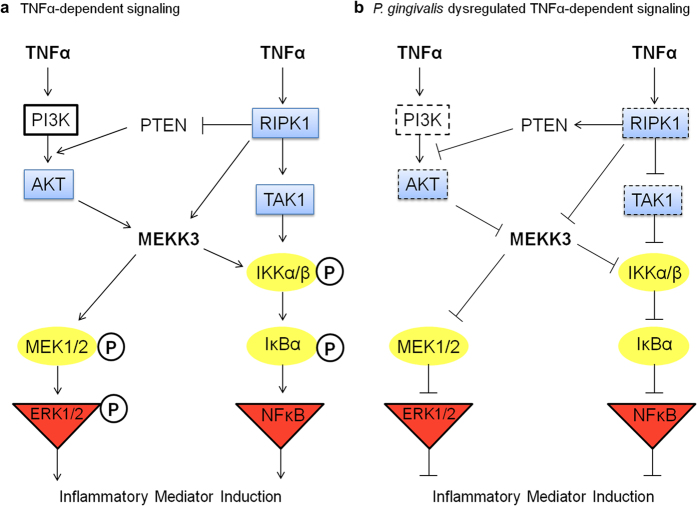
Model of *P. gingivalis-*induced attenuation of TNFα-dependent innate immune signaling responses. (**a**) Normal TNFα-dependent signal propagation is capable of proceeding through both AKT and RIPK1-dependent pathways, which culminates in the activation of either ERK1/2 or NFκB and thus promotes inflammatory mediator induction. RIPK1 and AKT can both interact with MEKK3, providing cross-talk for signal flux between these pathways. Additionally, RIPK1 indirectly activates AKT via its role in reducing expression of PTEN, a phosphatase that inhibits the PI3K/AKT pathway. During TNFα stimulation, signal flux can be monitored through these pathways by the detection of high phosphorylation status of MEK1/2, IKKα/β and IκBα. (**b**) Here we demonstrated that *P. gingivalis* dysregulates TNFα-dependent signaling via degradation of host cell RIPK1, TAK1 and AKT kinases. Degradation of host kinases would limit the required interactions of RIPK1 and AKT with MEKK3, in addition to IKK recruitment, effectively blunting ERK1/2 and NFκB activation and proinflammatory induction. Cross-talk between pathways would be reduced due to a lack of RIPK1 initiating an increase in PTEN levels, allowing for inhibition of AKT activity. Attenuated signal flux was observed within downstream signaling proteins (reduced phosphorylation of MEK1/2, IKKα/β and IκBα). Of note, Nakayama *et. al.* have reported that *P. gingivalis* infection impairs PI3K/AKT activity through degradation of an unidentified membrane protein, creating a secondary assault to the signaling capacity of the AKT pathway. Dashed boxes represent host proteins whose protein abundance and/or activity are disrupted by *P. gingivalis.* Circled proteins are internal signaling proteins whose phosphorylation status are reduced and those in triangles are terminal signaling proteins identified to have reduced phosphorylation and/or activity during *P. gingivalis* induced dysregulated TNFα signaling.

Cell signaling networks are a core component of the innate immune response and are necessary for initiation, amplification and long term pathway activation. Pathogen targeting of cell signaling proteins increases the potential to alter multiple cellular processes downstream of a single target, before signals diverge to additional responses. In this study we describe a novel pathogen evasion strategy that targets intracellular signaling via protease degradation of kinases essential to innate immunity. Selective targeting of the distinct kinases RIPK1, TAK1 and AKT, dampened *P. gingivalis* mediated immune responses while also attenuating paracrine TNFα signaling. Both ERK and NFκB pathway activation was reduced following TNFα stimulation of cells cocultured with *P. gingivalis*, revealing impairment of proinflammatory mediator induction and dysregulation of TNFα-dependent signaling (Fig. 7).

A number of bacterial pathogens have evolved mechanisms to interfere with NFκB signaling. Intervention of signal initiation or propagation, regulatory components, ubiquitination status, or transcription factor function have been described^34^. Specifically, microbial effectors have been identified to inhibit ubiquitin-mediated degradation of IκBα, modulate IKK complex function, acetylate MAPK, or block RelA nuclear translocation^34^. RIPK1 and TAK1 are essential kinases, which function downstream of TNF-R and TLR ligation, propagating essential host defense signals via scaffolding or proximity mediated phosphorylation events^35^. Studies using knockout cells have highlighted the central roles of RIPK1 and TAK1 in immune signaling, as NFκB, MAPK and IKK activation, are all abolished or severely attenuated in RIPK1^−/−^ or TAK1^−/−^ cells^7^,^8^,^36^,^37^,^38^, demonstrating the promiscuity of RIPK1 function towards other signaling pathways beyond NFκB (Fig. 7a). AKT is a serine/threonine kinase that is not a canonical member of the NFκB pathway but can modulate flux through the pathway (Fig. 7a). AKT is thought to exert a quantitative effect upstream of IKK activation, resulting in an amplification of the NFκB response^39^. Microarray data using an AKT inhibitor or siRNA established that AKT is required for a subset of NFκB-dependent genes but also affects the signal flux and kinetics of NFκB activation downstream of CD3/CD28 within T cells^39^.

Notably, in this study we identified significant decreases in TAK1 and AKT during *P. gingivalis* coculture, and protein level kinetics and Kgp dependency mimicked that of RIPK1, as reported previously^22^. Our results suggest that *P. gingivalis* dampens immune responses via severe reductions in RIPK1, TAK1, and AKT kinase levels, resulting in poor signal propagation downstream of PRR ligation, resulting in significant reductions in terminal ERK pathway activation. *P. gingivalis* has been reported to modulate ERK in gingival epithelial cells^17^, while a few reports have examined the ability of both bacterial and viral pathogens to target intracellular kinases. *Shigella* can dephosphorylate MAPK proteins^40^ and HIV has been reported to degrade RIPK1^41^, resulting in downstream functional consequences. Thus, targeting of RIPK1 has evolved for both bacterial and viral pathogens, although this is the first report demonstrating disruption of both RIPK1 and TAK1, providing an opportunity to maximize downstream effects in preventing inflammatory mediator induction. While we cannot exclude other Kgp-dependent inflammatory effects, such as degradation of coreceptors (CD14)^42^, our data imply a strong correlation between Kgp-dependent kinase degradation and host immune activation status.

Paracrine TNFα signaling is an important aspect of bacterial infections as it recruits active immune cells capable of secreting TNFα within the milieu, resulting in diverse effects on resident cells. Notably, we found that endothelial cells cocultured with bacteria failed to produce TNFα and were less capable of responding to TNFα, highlighting a dysfunction in TNFα paracrine function. Importantly, exogenously added TNFα remained biologically active in the presence of proteolytically active *P. gingivalis*, suggesting that Kgp does not cleave TNFα itself. The effects of Kgp on impairment of TNFα-dependent signaling were multi-factorial. We observed reduced activation of both ERK and NFκB pathways. Both IκBα and IKKα/β phosphorylation were examined due to the complex nature of NFκB signaling, where others have indicated that IκBα degradation may be rapid and robust, yet IKK activation contributes to resynthesis of IκBα, which may partially mask cell activation readouts^43^. The reduction in ERK and NFκB activation are likely results of reduced AKT or RIPK1 protein abundance, as both are upstream events (Fig. 7). Cells stimulated with *P. gingivalis* retained their ability to activate both p38 and JNK pathways normally, which is similar to studies examining nonfunctional RIPK1 or AKT^8^,^44^, suggesting that distinct downstream pathways exist and Kgp-mediated degradation effects are selective. Furthermore, RIPK1 can lower PTEN activity/expression, causing a derepression in PTEN-dependent AKT inhibition, thus generating a possible convergence between RIPK1 and AKT pathways upstream of MEKK3 as well (Fig. 7)^45^. Indeed, evidence of direct interactions of RIPK1 with AKT^46^ creates a scenario of bacterial-induced targeting of distinct host kinases due to proximity to one another. Disruption of RIPK1 or AKT protein levels and/or activity prevented immune mediator induction during TNFα stimulation, suggesting that pathogen intervention of either pathway can provide substantial effects on innate immune activation. The distinct role and place of MEKK3 within specific signaling pathways is not fully characterized and remains a focus of future studies.

Knockdown studies demonstrated that RIPK1 siRNA alone had a modest effect on impairment of signaling, however knockdown of RIPK1 and TAK1 resulted in an additive effect with increased immune signal pathway dysregulation. We surmise that the remaining pool of detectable TAK1 would be limited in its recruitment to the TNF-R complex because of its requirement for RIPK1. Recruitment of IKK can still proceed to some degree, due to the function of TRAF2, however IKK activation would be blocked during RIPK1-deficiency^38^. Interestingly, coculture with *P. gingivalis* alone impaired host cell signal activation to a higher extent compared to RIPK1 siRNA. We observed that coculture with *P. gingivalis* reduced RIPK1 protein levels more drastically than siRNA treatment, implying that siRNA treatment failed to reduce RIPK1 protein below a threshold level required for normal signal activation. Bacterial coculture did not reduce RIPK1 and TAK1 protein abundance to absolute zero, allowing for residual signaling potential to remain, as we observed some degree of response to TNFα stimulation. This suggests that other RIPK1-independent signaling pathways may remain functional, contributing to the observed levels of activation. We would propose that alterations to RIPK1 protein levels may also have repercussions on shifting the balance of pro-survival versus death-inducing functions of RIPK1. Inhibition of host cell death by limiting apoptosis or necroptosis could establish a more favorable scenario for the pathogen, however future studies will have to be performed to examine these consequences.

Others have demonstrated that specifically targeting RIPK1 and TAK1 offers maximal opportunities in disruption of cell signaling. A systems biology approach, using dynamic computational modeling, was used to investigate TNFα signal transduction to identify all branching pathways and the roles of each signaling member^47^. Among the 12 signaling proteins analyzed, the removal of the TAK1 complex (TAK1/TAB1/2) or RIPK1 produced the most noticeable down regulation of early, intermediate, and late induced genes during TNFα stimulation. Deletion of RIPK1 specifically displayed 50–70% impairment compared to the wild-type peak expression^47^, a result mimicked within our studies, as RIPK1 protein levels dictated the ability of host cells to respond to TNFα.

An important observation from our studies was that cells cocultured with *P. gingivalis* still possessed the ability to activate AKT, however the magnitude of activation was significantly lower due to the reductions in total AKT protein abundance (Fig. 7b), or possible effects on PI3K activity, considering our observations that PI3K protein abundance is similarly reduced in the presence of *P. gingivalis*. We also determined that incubation with a PI3K/AKT inhibitor resulted in reduced endothelial activation of NFκB in response to TNFα, suggesting AKT has a role in TNF-dependent responses within endothelial cells. *P. gingivalis* gingipain-dependent attenuation of PI3K/AKT signaling has been reported in gingival epithelial cells, however in contrast to our data, changes to AKT protein abundance following bacterial infection was not observed, suggesting strain-specific differences^48^. Furthermore, our results and additional reports suggest that wild-type strain 381 is more efficient at degradation of intracellular host proteins, yet strain 33277 is more effective at degradation of secreted cytokines^49^. Other pathogens, such as *Francisella,* also appear to interact with the host PI3K/AKT signaling pathway. Infection studies demonstrate that PI3K/AKT positively regulates the host NFκB response during *Francisella* infection, while also being host-protective^50^. Genome wide studies have however indicated pathogen suppression of PI3K signaling in human phagocytes by down-regulation of host cell PI3K/AKT expression^51^. Subversion of AKT signaling is not unique to *Francisella* and *P. gingivalis*, as uropathogenic *E. coli, S. aureus*, and *Aeromonas,* all inhibit AKT activation, mainly through the use of toxins^52^. Interestingly, *B. anthracis* inhibition of AKT activation occurs at the ERK/PI3K cross-talk^53^, lending support for our findings that *P. gingivalis* impairment of ERK activation likely proceeds in part through an AKT-dependent pathway (Fig. 7).

There are a number of possibilities by which Kgp gains access to intracellular host kinases for subsequent degradation. The majority of adherent bacteria actively invade endothelial cells, suggesting a highly efficient endocytic uptake that occurs at lipid raft regions^23^,^24^. These lipid rafts are known to serve as platforms for cellular signaling, functioning to cluster high concentrations of receptors and adaptor proteins^54^. Upon activation, TNF-R and AKT translocate to lipid rafts^55^,^56^, where they bind various signaling proteins, such as RIPK1 and TAK1, or PIP3, a phospholipid enriched within lipid rafts^57^, respectively, such that bacterial entry coincides with the location of recruited kinases/adaptors. Clustering of specific host cell signaling kinases may explain the mechanism by which Kgp targets only certain host proteins, such that compartmentalization of Kgp would concentrate and localize its activity, possibly explaining why distinct sets of proteins are degraded. Furthermore, gingipains are associated with outer membrane vesicles (OMV) that are formed following blebbing from the bacterial membrane, and thus have the potential to exert effects distant from the organism. It is possible that soluble or OMV-contained proteolytic activity plays an important role in intracellular proteolysis independent of intact bacteria, as both enter cells rapidly^58^,^59^. In support of this, we observed significant effects on host kinase protein levels during interactions with bacterial supernatants alone. Notably, Kgp is the most abundant protein within *P. gingivalis* OMVs within certain strains^60^, while these OMVs have been described to interact with intracellular PRR within non-phagocytic cells, such as the NOD2-RIPK2 signaling complex^61^. Finally, OMVs may fuse with the plasma membrane followed by delivery of vesicle contents, enabling Kgp interactions with cytosolic contents. Such a mechanism has been identified to occur within minutes in *Pseudomonas,* where OMV mediated delivery of virulence factors resulted in increased efficacy in altering host physiology as compared to purified protein^62^.

**Figure 8:**
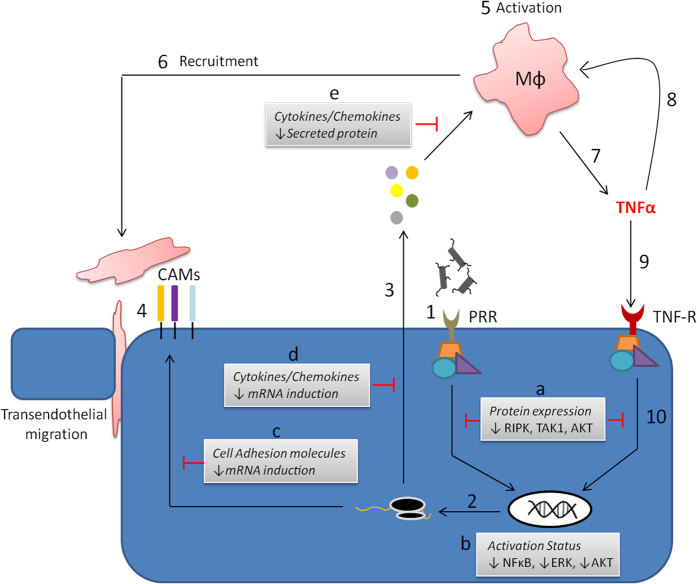
Model of *P. gingivalis* gingipain-driven subversion of innate immunity at the endothelium. Immune activation of the endothelium during microbial infection involves a number of critical events that are influential in facilitating resolution of infection. (1). Recognition of pathogens by the endothelium occurs through pattern recognition receptors, effectively driving specific genetic programs, leading to mRNA induction (2) and endothelial activation. This is primarily observed as secretion of cytokines and recruitment factors (3), in addition to the up regulation of cell adhesion molecules (4). Inflammatory mediator output activates (5) and recruits (6) immune cells to the site of the infection in order to help mediate clearance and recovery of the tissue, while cell adhesion molecules allow for enhanced transendothelial migration of professional immune cells into deeper tissues. Recruited inflammatory cells can produce large amounts of TNFα (7), which can act in an autocrine (8) or paracrine fashion (9). Endothelial responsiveness to TNFα functions as an amplification step to further activate the endothelium itself (10), while also promoting increased immune cell recruitment and/or activation due to further increases in inflammatory mediator output. We have demonstrated that *P. gingivalis* gingipains modulate endothelial innate immunity at several key points. Degradation of RIPK1, TAK1 and AKT kinases (**a**) results in attenuated responsiveness to TNFα. This was observed as reduced activation of NFκB, ERK and AKT pathways (**b**), in addition to impaired mRNA induction of both cell adhesion molecules (**c**) and secreted cytokines and chemokines (**d**). Analogous to TNFα-dependent responses, we propose that host immune kinase expressional changes (**a**) mediate reduced mRNA induction downstream of PRR-dependent responses to *P. gingivalis*. Significantly, as opposed to simply subverting the ability of the endothelial cell to properly induce inflammatory mediator secretion, gingipain activity can directly degrade already produced and secreted effectors (**e**).

To achieve proper immune activation at the endothelium during microbial infection specific genetic programs are induced, including cell adhesion molecules, secretion of cell activating cytokines, and recruitment factors (Fig. 8). We propose that *P. gingivalis* proteolytic activity disrupts innate immunity at the endothelium through a multi-pronged approach (Fig. 8), effectively blunting both professional and non-professional immune cell responses, which establishes a viable microenvironment for the microbe. Dysregulation of immune signaling via degradation of immune kinases or directed degradation of mature secreted factors impairs the quality of response, while specifically inhibiting paracrine TNFα-dependent responses (Fig. 8). This dysregulation of the innate immune amplification process may be a critical driving factor in pathogen persistence and chronic inflammation. An important distinction here is that *P. gingivalis* actively suppresses the immune response instead of acting to simply evade it, providing an environment where the ability of the host to respond to other pathogens or other inflammatory ligands would be jeopardized.

## Methods

### Reagents

Plasmids pDONR223-MAP3K7 (#23693) and PRK5-Myc RIPK1 (#44159) (RIPK1-myc) were purchased from Addgene.

### Human cell culture and growth conditions

Primary pooled human umbilical vein endothelial cells (HUVEC) (Lonza) were maintained in endothelial growth medium 2 (EGM-2). Experiments were performed with cells (passage 3–7) seeded in 6-well plates at a density of 3–5 × 10^5^ cells/well in EGM-2. Hela (ATCC CCL-2) and HEK293T cells (provided by Dr. Andrew Henderson, Boston University School of Medicine) were maintained in Dulbecco’s modified eagle medium (DMEM) supplemented with 10% fetal bovine serum (Hyclone) and penicillin-streptomycin supplementation (Invitrogen). Cells were seeded into 6-well plates at a density of 4–5 × 10^5^ cells/well. All cells were cultured at 37 °C and 5% CO_2_.

### Bacterial strains and growth conditions

*P. gingivalis* strains used in this study are included in Supplementary Table 2. Strains were maintained at 37 °C on 5% anaerobic blood agar (ABA) plates in an anaerobic atmosphere (10% H_2_, 10% CO_2_, and 80% N_2_) for 3 to 5 days. To evaluate the role of gingipains, isogenic mutant strains were used (ΔRgpA, ΔRgpA/B and ΔKgp; 33277 genetic background). To evaluate the role of fimbriae, we used strains DPG3 (major fimbriae mutant), MF1 (minor fimbriae mutant), and MFB (major and minor fimbriae mutant). For stimulation assays, ABA cultures were resuspended in bovine heart infusion broth (BHI, BD Biosciences) supplemented with 5 mg/mL yeast extract (BD Biosciences) and 10 μg/mL hemin (Sigma) and grown for approximately 18 hours. When necessary, bacterial mutant strains were grown in media supplemented with 5 μg/mL erythromycin (ΔRgpA, ΔRgpA/B, ΔKgp, DPG3, MFB) or 2 μg/mL tetracycline (MF1).

### *P. gingivalis* stimulation assays

Bacterial culture preparation and inhibition of gingipain activity was performed as previously described^22^. Host cell stimulation assays were performed in a humidified 37 °C, 5% CO_2_ incubator and were performed a minimum of 2 times, in triplicate per condition for each experiment. Cells were cocultured with cell culture medium (M) or live bacteria (ranging from multiplicity of infection (MOI) 10–100) for 1, 2, 3, 4, 6, or 24 hr. For some experiments, HUVEC were pre-incubated with the proteasome inhibitor MG132 (Sigma) for 2.5 hr (30 μM), cytochalasin D for 30 min (1 μg/mL), or the PI3K/AKT inhibitor Ly294,002 hydrochloride (Sigma) for 3 hr (25 μM), prior to coculturing with *P. gingivalis*. To assess alterations in host cell signaling following *P. gingivalis* coculture, some experiments consisted of restimulation with TNFα (10 ng/mL) for 5, 15, 30, 45, or 60 min. To monitor host cell mRNA expression following *P. gingivalis* coculturing, cells were left untreated or cocultured for 2 hr, followed by TNFα restimulation for 6 hr. Lastly, HUVEC were left untreated or cocultured (381 or 381/KYT-36 at a MOI 100) for 2 hr, followed by restimulation with TNFα for either 1 or 6 hr. Cell culture supernatants were collected to assess TNFα within the *in vitro* culture system.

### Sample collection and Western blot analysis

Whole cell lysate collection and Western blot analysis was performed as described previously^22^. Primary antibodies used include those to upstream signaling proteins: TAK1 (Cell signaling technology, CST), RIPK1 (BD), RIPK2 (Prosci), AKT (CST), and cIAP2 (Santa Cruz), downstream signaling proteins: ERK, JNK, p-38, IκBα, ubiquitin and IKKβ (all CST), phospho-proteins: P-AKT, P-ERK, P-JNK, P-p38, P-RAF1, P-MEK1/2, P- IκBα, and P-IKKβ (all CST), protein tags: myc (CST), FLAG (Sigma), or GAPDH (CST). For all figures, the membranes depicted are representative of a minimum of three independent membranes.

Quantification of Western blot protein signal intensity was determined using Image J software with samples from multiple experiments. The numbers of membranes used for calculation of protein abundance (relative density units; R.D.U.) is detailed in each respective figure legend. Data is represented as the mean + SEM of the target protein (P-ERK, P-JNK, P-p38, AKT, P-AKT, RIPK1, TAK1, P-IκBα, P-IKKα/β), relative to loading control (ERK or GAPDH). For tracking changes to host cell protein abundance in response to *P. gingivalis* infection alone, values were normalized to media control. When monitoring changes to innate immune signaling responses following RIPK1 siRNA, RIPK1 + TAK1 siRNA, or the effects of bacterial coculuture in a RIPK1 knockdown, cells treated with negative control siRNA were set = 1 R.D.U. Calculation of P-IKKα/β induction was performed by subtracting +TNFα sample from −TNFα sample for each respective condition.

### Gingipain effects on host cell secreted factors

To determine if coculturing with *P. gingivalis* resulted in alterations to host cell cytokine/chemokine secretion and/or detection, HUVEC were cocultured with strains 381, 33277, ΔRgpA, ΔRgpA/B, ΔKgp, or with strain 381 pre-incubated with gingipain inhibitors KYT-1, KYT-36, KYT-1/36, or TLCK for 24 hr. Cell culture supernatants were collected and analyzed via ELISA for detection of IL-8, IL-6 or MCP-1 with ELISA kits according to the manufacturer’s instructions (BD Biosciences).

### Host cell target degradation and efficiency

To assess the selective targeting and efficiency of gingipain-specific degradation of host cell proteins, *P. gingivalis* strain 381 was grown overnight in BHI broth for approximately 20 hr, until growth reached saturation (OD_660_ = >1). Optical densities were recorded and cultures were adjusted with BHI broth to equal OD_660_ = 1. Bacteria were pelleted for 10 min at 8000 rpm at room temperature. Supernatants were collected and passed through 0.22 μM filter to remove contaminating bacteria, where absence of bacteria was confirmed with plating on ABA plates. Initial supernatants (1x) were then diluted 1.35, 2, 4, 8, 16, or 32x which corresponded to supernatants containing Kgp-specific (Kgp-X) activities of 1.44, 2.07, 4.26, 8.04, 14.43, 22.97, and 33.75 mOD/min, respectively, as measured in triplicate. The range of Kgp-X containing supernatants were added to HUVEC cultures and incubated for 2 hr at 37 °C and 5% CO_2_. Whole cell lysates were collected and changes in protein levels to host cell RIPK1, TAK1 and AKT were monitored via Western blot analysis. Densitometry was performed on a minimum of three independent membranes to calculate percent protein degradation relative to media treated samples (set as 100% expression). Graph pad prism was used to plot percent degradation of each respective protein versus bacterial Kgp-X activity contained within each condition, allowing for calculation of proteolytic activity required to achieve 50% RIPK1 or TAK1 degradation. Coculturing with *P. gingivalis* 381 at an MOI 100 was included as a base measure of protein degradation under *in vitro* conditions.

### Molecular tagged TAK1 vector cloning and expression

Generation of an N-terminal FLAG-tagged TAK1 construct (TAK1-FLAG) was created using pDONR223-MAPK37 (Addgene) as a PCR template to amplify the TAK1 coding region with Phusion high fidelity polymerase (New England Biolabs). A start codon, Kozak sequence, FLAG tag and restriction sites (XhoI and NotI) were added to the TAK1 sequence using the following primers (5′-TTTTCTCGAGGCCACCATGGATTACAAGGATGACGACGATAAGTCTACAGCCTCTGCCGCCTCC, 3′- TTTTGCGGCCGCTCATGAAGTGCCTTGTCGTTTCTGCTGCTG). The PCR product was digested and ligated with T4 DNA ligase into a CIP-treated pGAW1004 (provided by Dr. Gregory Wasserman, Boston University School of Medicine) vector using restriction sites XhoI and NotI. The expression vector was transformed using *E. coli* TOP10 (Life Technologies) competent cells and transformants selected on LB plates containing Amp^100^. Transformants were screened via restriction analysis and sequencing of isolated plasmid DNA confirmed proper sequence. Overexpression in Hela cells was performed and Western blot analysis confirmed expression with either an anti-FLAG or anti-TAK1 antibody.

### Host cell siRNA knockdown

For knockdown of host cell protein expression, HUVEC were seeded in 6-well plates at 2–5 × 10^5^ cells/well and incubated for 5 hr. Transfection of 100 pmol stealth siRNA specific for either RIPK1 or TAK1 (Life Technologies) was added with RNAiMAX transfection agent (Invitrogen) diluted in Opti-MEM reduced serum media (Gibco) and incubated for 20 hr. Negative control samples were treated with equivalent negative control siRNA (Life Technologies). Stimulation experiments were then carried out with addition of media, TNFα, or *P. gingivalis* strain 381. For restimulation experiments, initial addition of media or *P. gingivalis*, followed by secondary addition of media or TNFα was performed. Efficient knockdown of target proteins was confirmed via Western blot analysis for each independent experiment.

### L929 TNFα bioactivity assays and determination of cell culture TNFα during *P. gingivalis* infection

To determine if incubation of *P. gingivalis* with TNFα-stimulated HUVEC cultures affected the bioactivity of TNFα, cells left untreated or cocultured with *P. gingivalis* at a MOI 100 (strain 381 or 381 pretreated with 10 μM KYT-36) were incubated for 2 hr, followed by restimulation with TNFα for 1 or 6 hr. Cell culture supernatants were collected and assayed for TNFα by L929 cytotoxicity bioassay and ELISA. The bioassay consisted of L929 cells seeded at 3.5 × 10^4^/well in 96-well plates and cultured overnight at 37 °C and 5% CO_2_. Actinomycin D (Sigma) was added (50 μl of 4 μg/mL stock), followed by the addition of supernatants (2 μl) containing unknown TNFα concentrations. After 24 hr, 10 μl of 5 mg/mL MTT solution (Sigma) was added to each well and incubated for 4 hr, followed by addition of MTT lysing solution. Absorbance was read at 570–650 nm. Serial dilutions of recombinant TNFα (0.625–0.003 ng/mL, BD Biosciences) were used to determine the quantity of bioactive TNFα within experimental samples by establishing a dose response curve relating percent cytotoxicity to concentrations of TNFα using Graph pad prism software. Wells containing media alone was used as the 100% viable control. Percent cytoxicity was calculated by A_100% viable control cells_−A_test wells_/A_100% viable control cells_ X 100. Experiments were performed twice in triplicate.

### RNA isolation and quantitative PCR

RNA and protein were isolated with TRIzol (Thermo). Subsequent processing/isolation of RNA and protein were carried out according to the manufacturer’s instructions. RNA sample purity and concentration were assessed by measuring A260/280 spectrophotometrically and were stored at −80 °C. cDNA was synthesized using high capacity cDNA reverse transcription kit (Applied Biosystems) following manufacturer’s instructions. All qPCR primers were purchased from Applied Biosystems and represented probes targeting RIPK1, TAK1, AKT, M-CSF, GM-CSF, IL-8, IL-6, MCP-1, Mip3α, VCAM-1, ICAM-1, cIAP2, and β-actin. The real-time qPCR reactions were performed using Taqman gene expression assays (FAM dye-labeled MGB probes) and Taqman gene expression master mix (2x) (Applied Biosystems) according to manufacturer’s instructions. The real-time qPCR reactions were performed on a Step one plus real-time PCR system (Applied Biosystems). mRNA expression data were normalized to the levels of β-actin as an internal control. Western blot analysis on the stored cell lysates was performed on protein isolated from the same well as the sample used for qPCR, in order to ensure expected changes to RIPK1/TAK1 occurred.

### NFκB reporter activity

To determine the effects of *P. gingivalis* infection on TNFα-dependent innate immune signaling responses, an NFκB reporter was used as a measure of cellular activation status. HEK293T cells were seeded at 2 × 10^5^ cells/well in 6-well plates and allowed to rest for 24 hr. Cells were transfected for 24 hr with 1 μg NFκB-Luciferase reporter plasmid containing the luciferase gene and three tandem NFκB transcription response elements (provided by Dr. Andrew Henderson, Boston University School of Medicine), using Lipofectatime 2000 (Applied Biosystems). Concurrently, a β-galactosidase control vector (pSV-β-Gal, Promega) was used as a transfection control. Cells were left untreated, cocultured with *P. gingivalis* 381 MOI 100, or 381 pre-incubated with 10 μM KYT-36 for 2 hr. Restimulation with 10 ng/mL TNFα was allowed to proceed for 6 hr, followed by whole cell lysate collection in 200 μl reporter lysis buffer (Promega). NFκB activity for each condition was calculated by measuring the luminescence using a luciferase assay system (Promega), per manufacturer’s directions.

### Gingipain activity assays

The arginine- and lysine-specific protease activities of whole cell-depleted bacterial supernatants were measured using the substrate hydrolysis of Nα-benzoyl-DL-arginine 4 *p*-nitroanilide (Sigma) or N-Tosylglycl-L-prolyl-L-lysine-4-nitroanilide (Sigma), as previously described^22^. Each analysis was performed in triplicate and activities are represented as mOD/min.

### Overexpression studies

To establish if overexpression of RIPK1 and TAK1 could repair the cellular responsiveness to TNFα following *P. gingivalis* coculturing, Hela cells were transfected with either empty vector (pGAW1004), or 1 μg RIPK1-myc + 1 μg TAK1-FLAG, using Lipofectamine 2000 transfection agent (Life Technologies). Transfection was allowed to proceed for 20 hr, followed by cells being left untreated or cocultured with *P. gingivalis* 381 MOI100 for 2 hr and then restimulation with 10 ng/mL TNFα. Western blot analysis confirmed overexpression of target proteins and examination of innate immune signaling was monitored via detection of P-IKKα/β.

### Statistics

All values are expressed as the mean value + standard error of the mean (SEM). Experiments were performed a minimum of two times in triplicate. Calculation of Western blot densitometry data utilized a range of individual membranes (n = 3–5), detailed within each figure legend. Statistical analysis included student’s t-test, one-way ANOVA or two-way ANOVA, when applicable, as listed within each figure legend. A p value < 0.05 was considered significant.

## Additional Information

**How to cite this article**: Barth, K. and Genco, C. A. Microbial Degradation of Cellular Kinases Impairs Innate Immune Signaling and Paracrine TNFα Responses. *Sci. Rep.* **6**, 34656; doi: 10.1038/srep34656 (2016).

## Supplementary Material

Supplementary Information
