# Supplementary material for: Microbial Degradation of Cellular Kinases Impairs Innate Immune Signaling and Paracrine TNFα Responses
Source: Sci Rep. 2016 Oct 4;6:34656. doi: 10.1038/srep34656 (PMC5048168; doi:10.1038/srep34656)
Supplement: Supplementary Information [file srep34656-s1.pdf]

Microbial Degradation of Cellular Kinases Impairs Innate Immune Signaling and Paracrine TNFα Responses

Kenneth Barth and Caroline Attardo Genco

Supplemental Table 1. Endothelial mRNA induction during *P. gingivalis* and TNFα stimulation

Proinflammatory Mediator	*P. gingivalis*-dependent induction[#]	*P. gingivalis* + TNFα	TNFα-dependent Induction (fold)*
GM-CSF	440 ± 131.8	2393 ± 736.7	5.4
IL-8	12.9 ± 1.2	38.9 ± 5.6	3.0
MCP-1	6.0 ± 1.0	21.9 ± 4.0	3.7
M-CSF	2.0 ± 0.1	13.6 ± 1.6	6.8
Mip3α	80.7 ± 30.0	301.6 ± 37.4	3.7
VCAM-1	12.2 ±2.5	104.4 ± 10.7	8.6
IL-6	3.9 ± 0.6	6.8 ±1.2	1.7

Mean mRNA fold change ± SEM of 381 stimulated HUVEC / untreated (media)

* Mean mRNA fold change ± SEM of 381 stimulated HUVEC + TNF/ 381 stimulated HUVEC - TNF

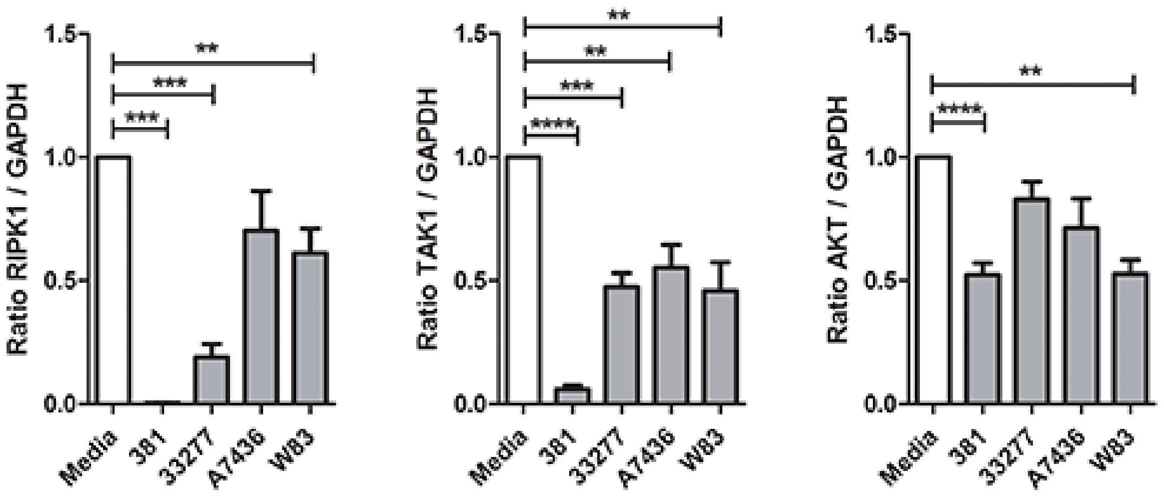

Supplemental Figure 1. Degradation of RIPK1, TAK1, and AKT occurs across wild-type *P. gingivalis* strains. HUVEC were cocultured with wild-type strains 381, 33277, A7436, or W83 for 2 hr. Whole cell lysates were analyzed for RIPK1, TAK1, AKT, or GAPDH expression via Western blot analysis. Relative density units was calculated for **(a)** RIPK1, **(b)** TAK1, and **(c)** AKT, setting untreated cells (media) = 1, using densitometry of three independent membranes. ****$p<0.0001$, ***$p<0.001$, **$p<0.01$, *$p<0.05$, using a Student's t-test comparing media to each respective wild-type strain.

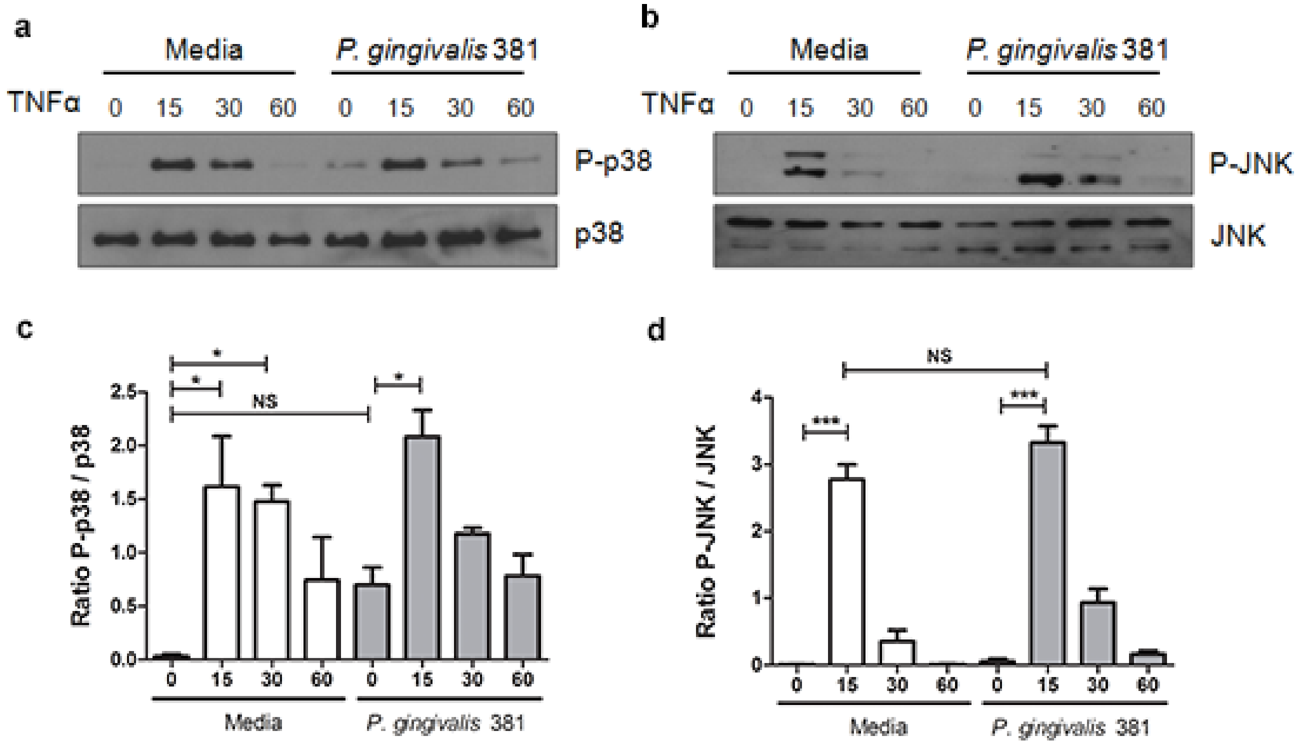

Supplemental Figure 2. Coculture of *P. gingivalis* with endothelial cells fails to alter TNFα-dependent p38 or JNK activation. Cells were either left uninfected (media) or cocultured (strain 381 MOI 100) for 2 hr at which time cells were restimulated with TNFα (10 ng/mL) for 15, 30, or 60 min. Whole cell lysates were examined for expression of **(a)** P-p38, or **(b)** P-JNK. The ratio of **(c)** P-p38 to total p38 expression or the ratio of **(d)** P- JNK to total JNK expression was quantified using densitometry from four independent Western blots. ***p<0.001, *p<0.05, NS=not significant, one-way ANOVA with Bonferroni post test.

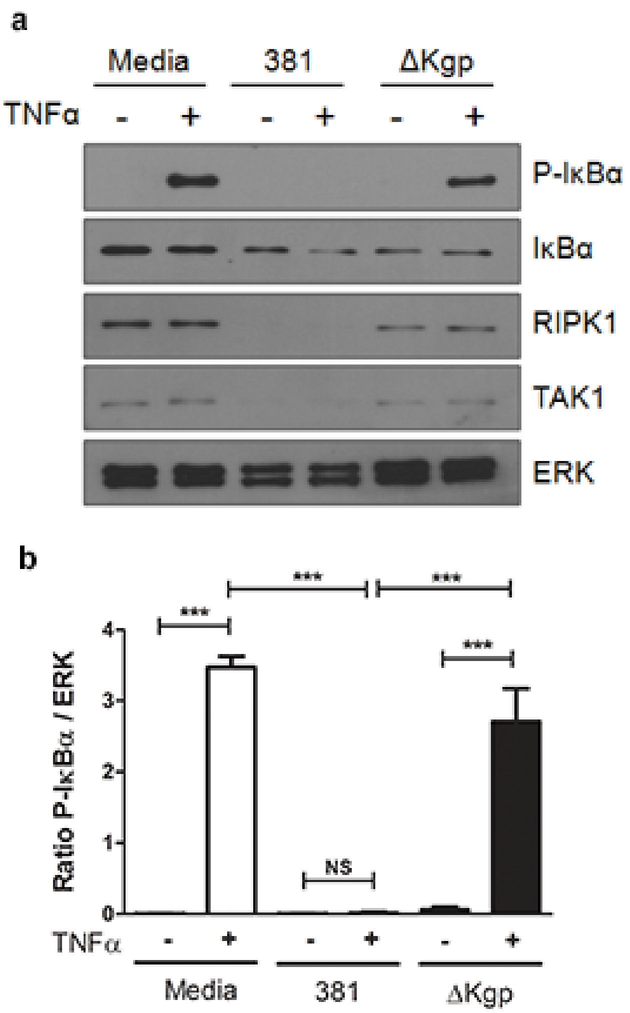

Supplemental Figure 3. *P.* gingivalis-driven impairment of TNFα-dependent signaling no longer occurs during ΔKgp coculture. (a) HUVEC were left untreated (media) or cocultured with MOI 100 *P. gingivalis* 381 or ΔKgp for 2 hr, followed by TNFα restimulation (10 ng/mL) for 60 min. Protein expression of phosphorylated and non-phosphorylated IκBα was measured via Western blot analysis. **(b)** The ratios of P-IκBα/ERK were calculated using densitometry (n=4). Values represent the mean + SEM, one-way ANOVA with Bonferroni post test. ***p<0.001, NS=not significant.

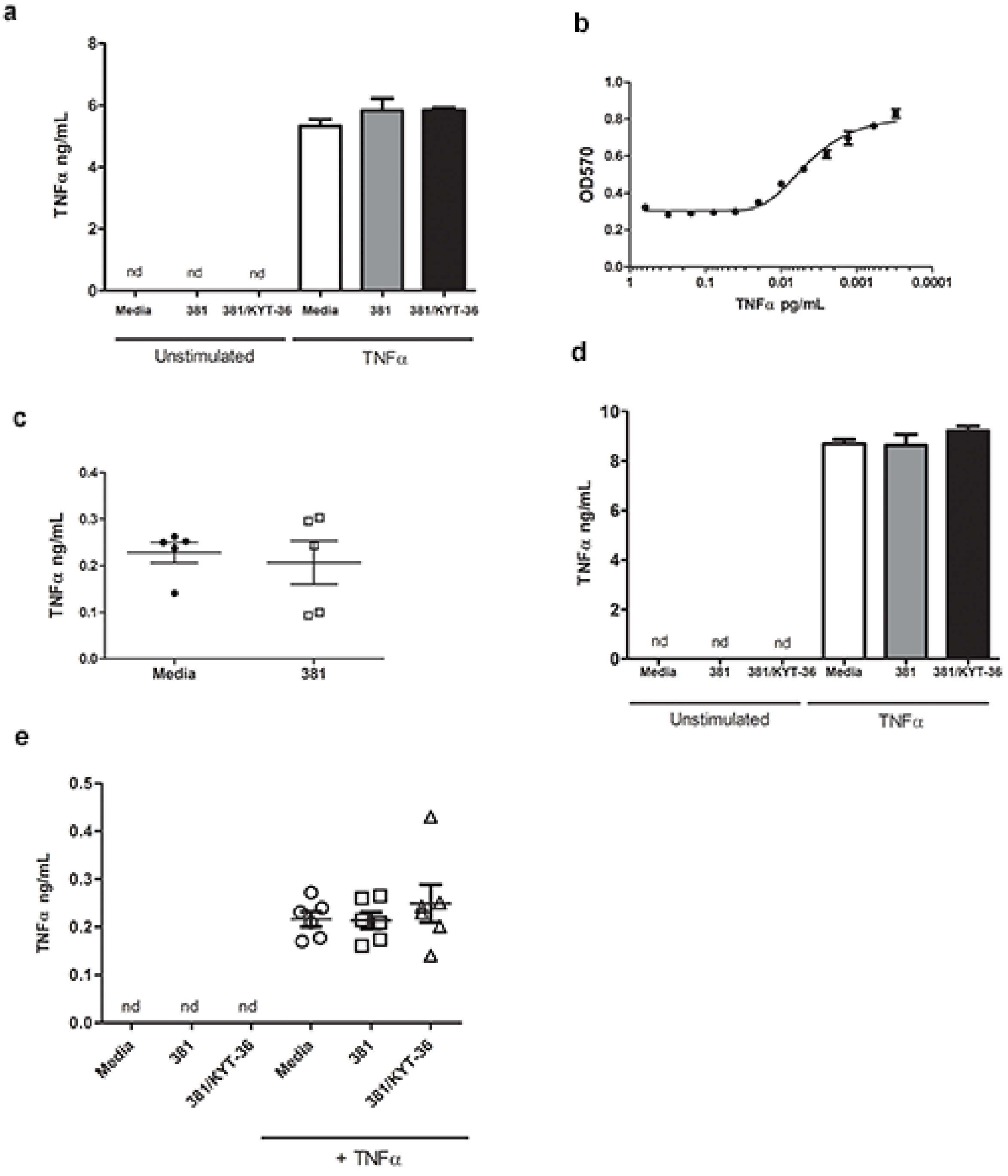

Supplemental Figure 4. Exogenously added TNFα is not degraded and remains bioactive during coculturing with *P. gingivalis.* **(a)** HUVEC were left untreated or cocultured with *P. gingivalis* MOI 100 (381 or 381 pretreated with 10 μM KYT-36) for 2 hr. Cultures were then left unstimulated or stimulated with TNFα (10 ng/mL) for 60 min. Cell culture supernatants were collected and assayed for TNFα levels by ELISA (n=3). **(b,c)** 2μl of each supernatant recovered from **(a)** was further used to measure the bioactivity of TNFα remaining under each condition by monitoring L929 cytotoxicity. **(b)** A standard curve for % cell viability was established using a range of TNFα (0.625-0.0003 ng/mL), allowing for the **(c)** calculation of bioactive TNFα remaining during 60 min coculturing with *P. gingivalis* (n=5). Nd= not detectable. **(d,e)** The amount of detectable and bioactive TNFα was measured as in **(a-c)**, however coculturing with *P. gingivalis* was extended to 6 hr.

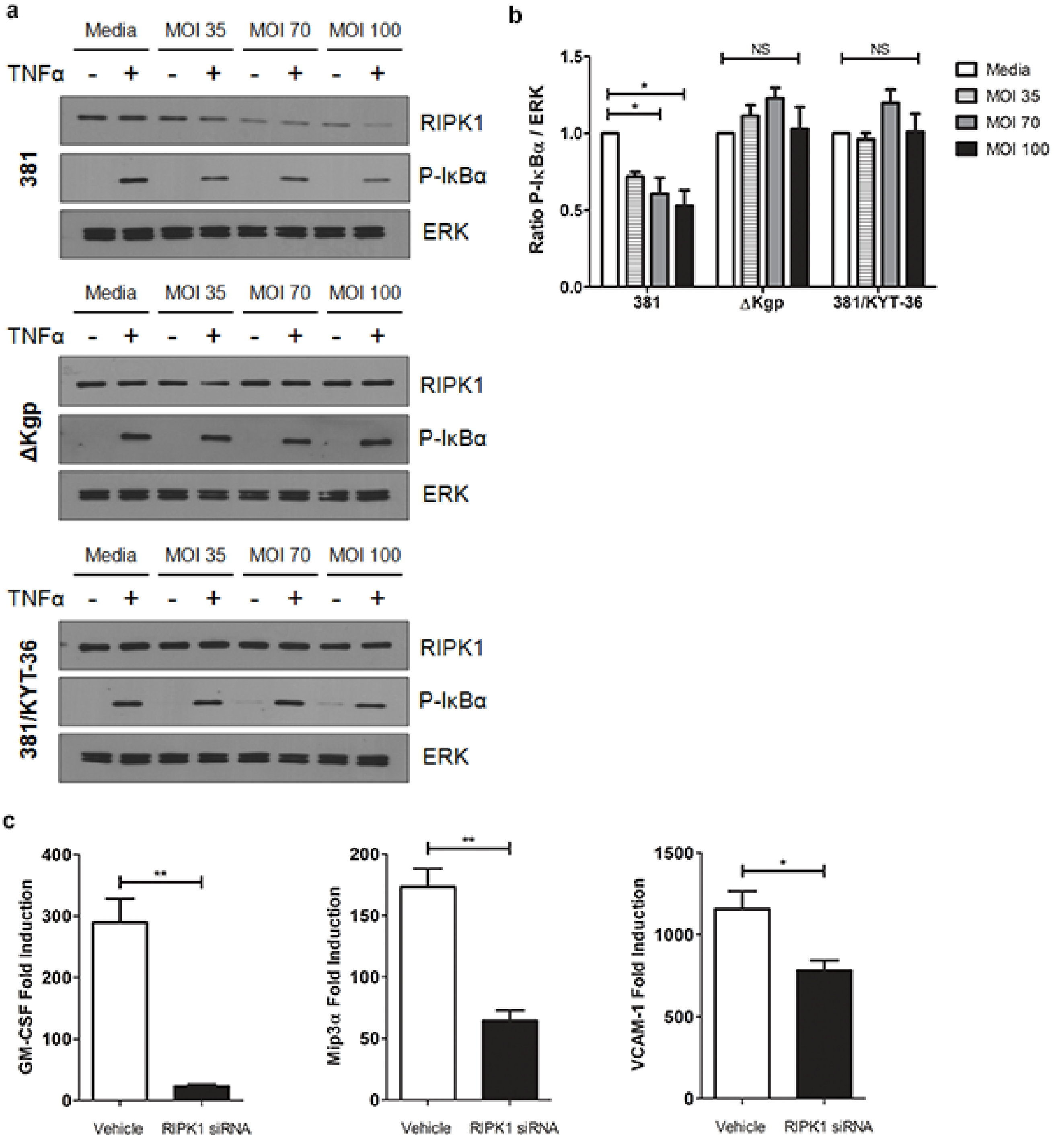

Supplemental Figure 5. Endothelial TNFα-dependent signaling and inflammatory mediator induction requires RIPK1 signaling. (a,b) HUVEC were left untreated or cocultured with *P. gingivalis* (381, ΔKgp, 381/KYT-36) at varying MOIs (35, 70, 100) for 2 hr. **(a)** RIPK1 and P-IκBα expression was monitored via Western blot analysis. **(b)** Densitometry (n=3) was used to calculate the ratio of P-IκBα /ERK. *p<0.05, NS=not significant, one-way ANOVA with Bonferroni post test comparing the effect of MOI for each strain. **(c)** HUVEC were transfected with either negative control or RIPK1 specific siRNA for 20 hr, followed by coculturing with *P. gingivalis* 381 MOI 100 for 2 hr and then restimulation with TNFα (10 ng/mL) for 6 hr. Immune activation was monitored via qPCR expression analysis. **p<0.01, *p<0.05, student's t-test.

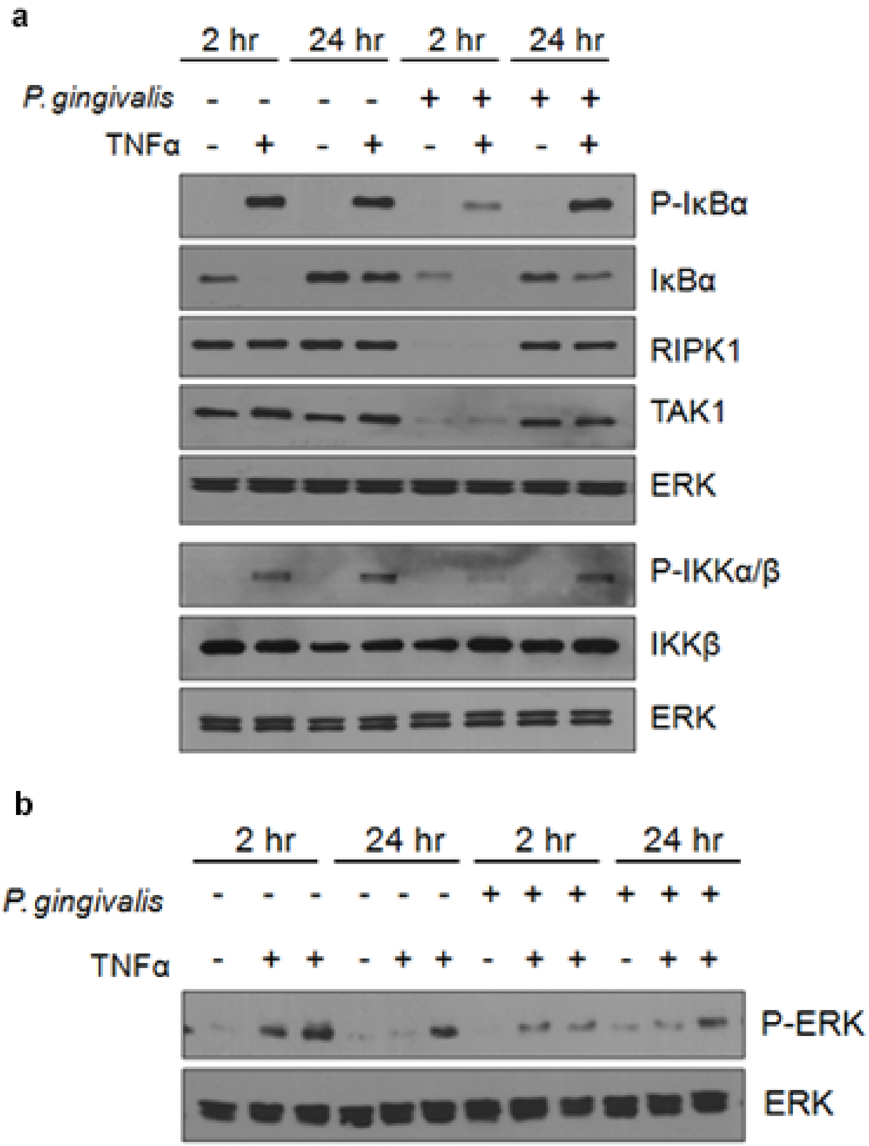

Supplemental Figure 6. Impaired cellular signaling correlates with both RIPK1 and TAK1 protein abundance. HUVEC were left untreated or cocultured with 381 for 2 or 24 hr, followed by TNFα restimulation for 60 min (top panel), 15 min (bottom panel) **(a)**, or 5 and 15 min **(b)**. Whole cell lysates were examined for endothelial responsiveness to TNFα by measuring activation status of IκBα, IKKα/β, and ERK-specific pathways.

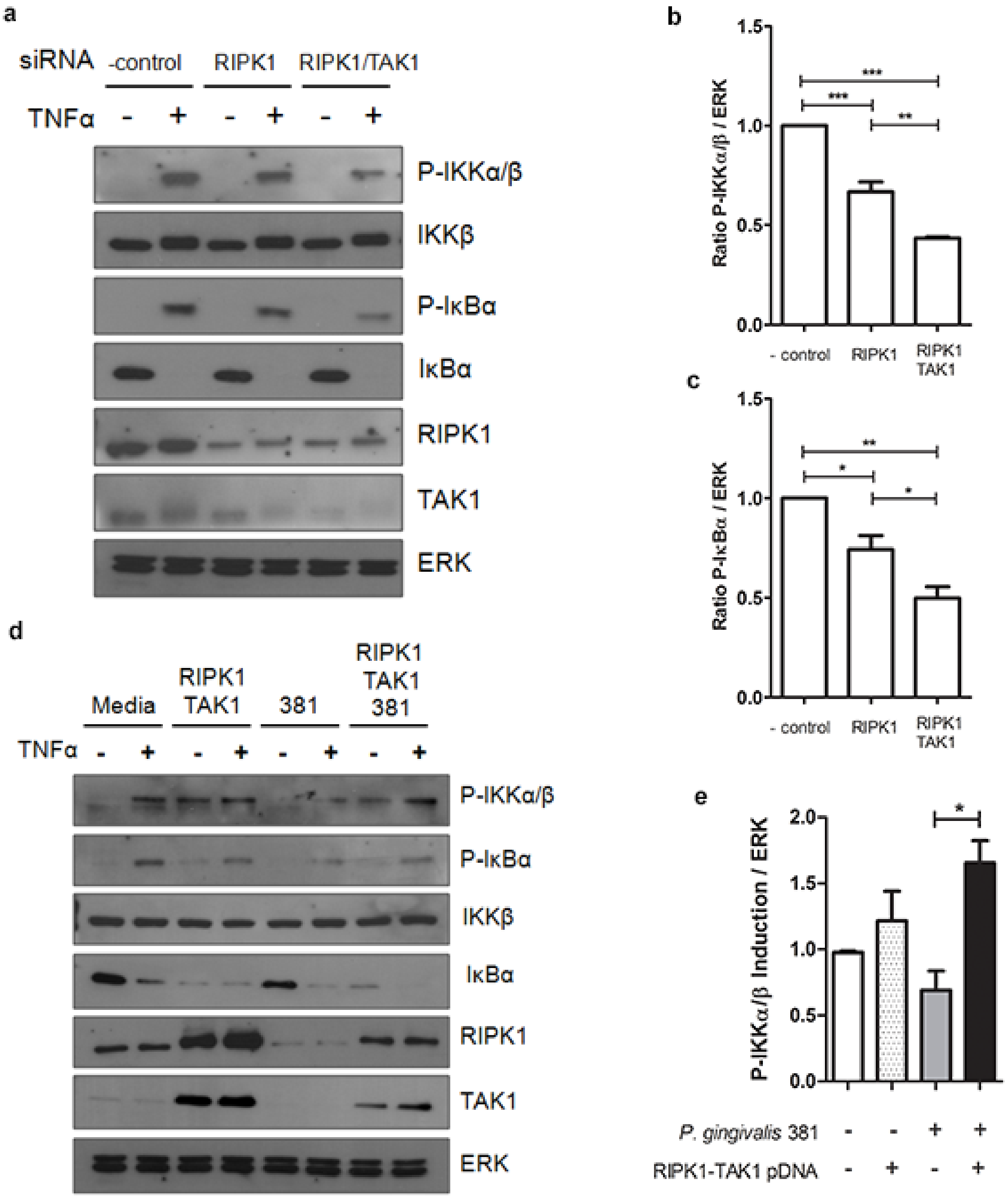

Supplemental Figure 7. RIPK1 and TAK1 protein abundance is required for optimal innate immune responses to TNFα. (a-c) HUVEC were transfected with either negative control or RIPK1 specific siRNA for 20 hr, followed by stimulation with TNFα (10 ng/mL) for 15 min. **(a)** Whole cell lysates were examined via Western blot analysis to determine activation status of the cells and to confirm efficient knockdown of RIPK1 and TAK1. Densitometry of independent membranes (n=3) was used to determine **(b)** P-IKKα/β and **(c)** P-IκBα expression. ***p<0.001, **p<0.01, *p<0.05, one-way ANOVA with Tukey's post test. **(d,e)** Hela cells were transfected with empty vector (-) or 1 µg RIPK1-myc + 1 µg TAK1-FLAG. After 20 hr, cells were left uninfected or cocultured with *P. gingivalis* 381 MOI 100 for 2 hr, followed by restimulation with TNFα (10 ng/mL) for 15 min. **(d)** Whole cell lysates were examined via Western blot analysis to confirm overexpression of RIPK1 and TAK1 signaling proteins, in addition to TNFα-dependent activation status. **(e)** P-IKKα/β induction was calculated by subtracting P-IKKα/β signal of + TNFα treated from - TNFα treated samples for each condition (n=3). Media control samples with empty vector was set = 1 R.D.U. *p<0.05 one-way ANOVA with Tukey's post test.

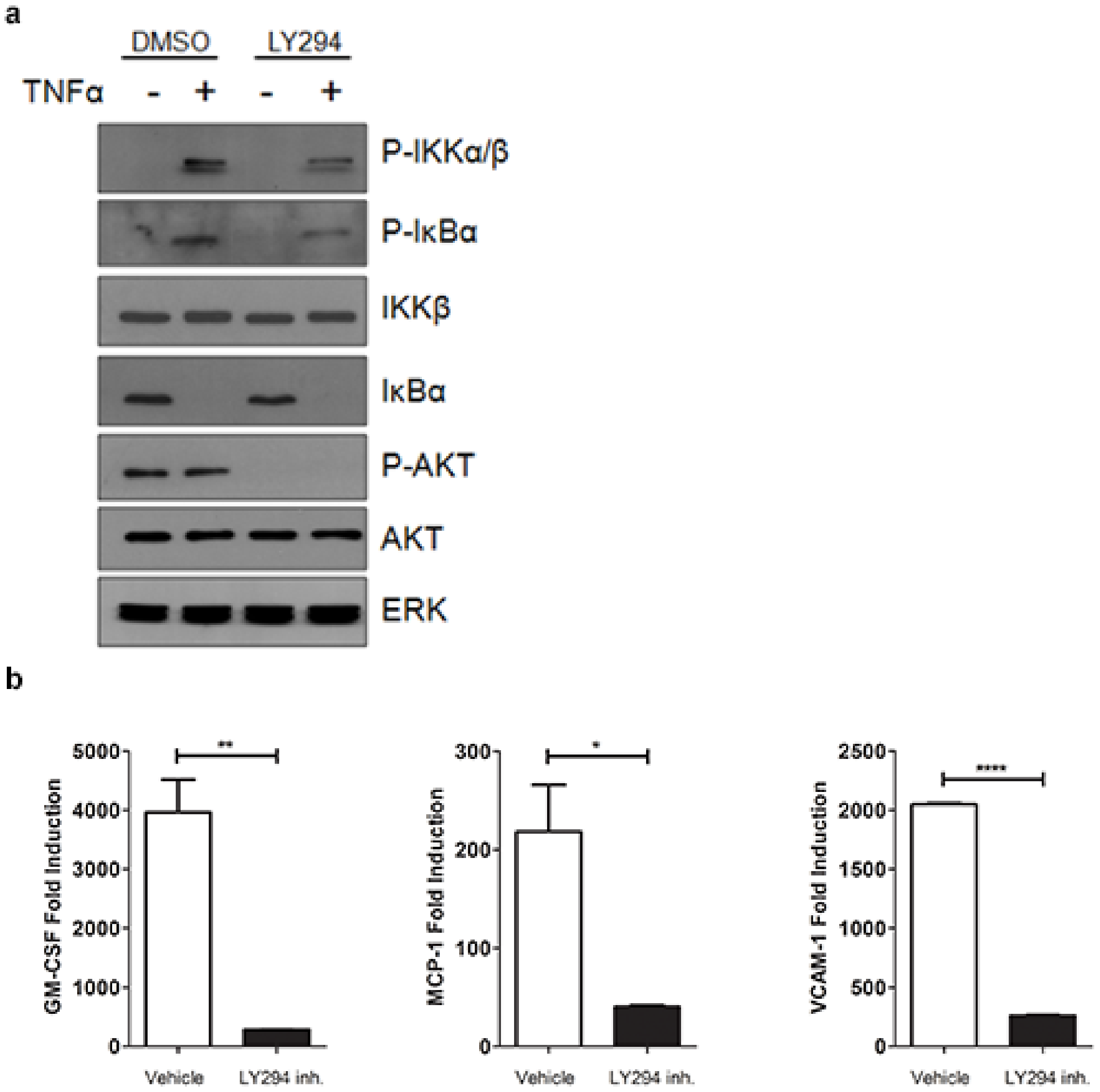

Supplemental Figure 8. AKT is required for maximal endothelial cellular responsiveness to TNFα. (a) HUVEC were pretreated with vehicle control (DMSO) or LY294 inhibitor (25 µM) for 3 hr, followed by stimulation with TNFα (10 ng/mL) for 15 min. Whole cell lysates were examined via Western blot analysis to determine TNFα-dependent activation status. **(b)** HUVEC were pretreated with Ly294 inhibitor (25 µM) for 1 hr, followed by coculturing with *P. gingivalis* 381 MOI 100 for 2 hr and restimulation with TNFα for 6 hr. Immune activation was monitored via qPCR expression analysis. ****$p<0.0001$, **$p<0.01$, *$p<0.05$, student's t-test.

Supplemental Table 2. Bacterial strains used in this study.

***P. gingivalis* strain**	**Characteristics (Genetic background)**	**Source**
381	Wild type	Laboratory Collection
DPG3	Major fimbriae mutant (381)	(1)
MF1	Minor fimbriae mutant (381)	(2)
MFB	Major and minor fimbriae mutant (381)	(2)
ATCC 33277	Wild type	Laboratory Collection
ΔRgpA	*rgpA* mutant (33277)	(3)
ΔRgpA/B	*rgpA*- and *rgpB*- mutant (33277)	(4)
ΔKgp	*kgpA* mutant (33277)	(3)
A7436	Wild type	Laboratory Collection
W83	Wild type	Laboratory Collection

1. Malek, R. *et al.* Inactivation of the Porphyromonas gingivalis fimA gene blocks periodontal damage in gnotobiotic rats. *J Bacteriold* **176,** 1052-1059 (1994).
2. Takahashi, Y., Davey, M., Yumoto, H., Gibson, F.C. 3rd & Genco, C.A. Fimbriae-dependent activation of pro-inflammatory molecules in Porphyromonas gingivalis infected human aortic endothelial cells. *Cell Microbiol* **8**, 738-757 (2006).
3. Park, Y. & Lamont, R.J. Contact-dependent protein secretion in Porphyromonas gingivalis. *Infect Immun* **66**, 4777-4782 (1998).
4. Nakayama, K., Kadowaki, T., Okamoto, K. & Yamamoto, K. Construction and characterization of arginine-specific cysteine proteinase (Arg-gingipain)-deficient mutants of Porphyromonas gingivalis. Evidence for significant contribution of Arg-gingipain to virulence *J Biol Chem* **270**, 23619-23626 (1995).
